# A survey of aquatic macroinvertebrates in a river from the dry corridor of Nicaragua using biological indices and DNA barcoding

**DOI:** 10.1002/ece3.9487

**Published:** 2022-11-05

**Authors:** Bryant H. Mendoza‐Ramírez, Lucía Páiz‐Medina, Thelma Salvatierra‐Suárez, Nelvia Hernández, Jorge A. Huete‐Pérez

**Affiliations:** ^1^ Molecular Biology Center University of Central America, UCA Managua Nicaragua; ^2^ Institute of Interdisciplinary Research in Natural Sciences University of Central America, UCA Managua Nicaragua

**Keywords:** aquatic macroinvertebrates, ASAP, BIN, biological index, BMWP, DNA barcoding, GMYC, IBF, PTP, species delimitation

## Abstract

Aquatic macroinvertebrates are widely used as indicators for water quality assessment around the world. Modern strategies for environmental assessment implement molecular analysis to delimitate species of aquatic macroinvertebrates. Delimitation methods have been established to determine boundaries between species units using sequencing data from DNA barcodes and serve as first exploratory tools for taxonomic revisions. This is useful in regions such as the neotropics where aquatic macroinvertebrate habitats are threatened by human interference and DNA databases remain understudied. We asked whether the biodiversity of aquatic macroinvertebrates in a stream in Nicaragua, within the Central American Dry Corridor, could be characterized with biological indices and DNA barcoding. In this study, we combined regional biological indices (BMWP‐CR, IBF‐SV‐2010) along with distance‐based (ASAP, BIN) and tree‐based (GMYC, bPTP) delimitation methods, as well as nucleotide BLAST in public barcode databases. We collected samples from the upper, middle, and low reaches of the Petaquilla river. The three sites presented excellent water quality with the BMWP‐CR index, but evidence of high organic pollution was found in the middle reach with the IBF‐SV‐2010 index. We report a total of 219 COI sequences successfully generated from 18 families and 8 orders. Operational taxonomic units (OTUs) designation ranged from 69 to 73 using the four methods, with a congruency of 92% for barcode assignation. Nucleotide BLAST identified 14 species (27.4% of barcodes) and 33 genera (39.3% of barcodes) from query sequences in GenBank and BOLD system databases. This small number of identified OTUs may be explained by the paucity of molecular data from the Neotropical region. Our study provides valuable information about the characterization of macroinvertebrate families that are important biological indicators for the assessment of water quality in Nicaragua. The application of molecular approaches will allow the study of local diversity and further improve the application of molecular techniques for biomonitoring.

## Graphical abstract

A survey of aquatic macroinvertebrates in Nicaragua using biological indices and DNA barcoding. We obtained excellent water quality in one site, high congruence between tree‐based and similarity‐based techniques, and reported 69–73 operational taxonomic units present in a river from the Central American Dry Corridor.

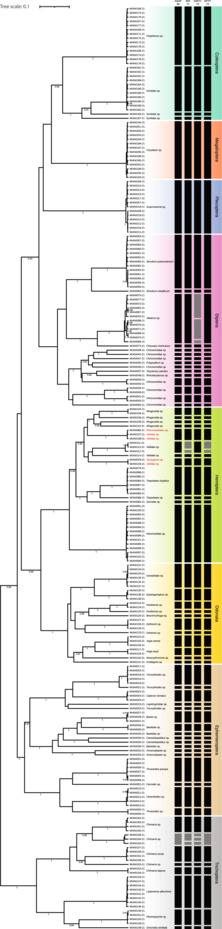

## 1 INTRODUCTION

Aquatic macroinvertebrates are the most widely used organisms in freshwater biomonitoring (Bonada et al., 2006). Biomonitoring indices often must rely on identification to family level since identification to species level is affected by problems associated with morphological complexity, condition of the specimens, and physiological aspects, such as size and life stages (Baird & Sweeney, 2011; Packer et al., 2009). However, using the lowest possible taxonomic level provides more information about specific environmental stressors (Lenat & Resh, 2001). In the Neotropical region, one of the most biologically diverse ecosystems of the world, aquatic habitats are threatened by human interference and aquatic macroinvertebrate diversity remains understudied (Bowles & Courtney, 2018). Molecular approaches could accelerate aquatic macroinvertebrates exploration in the neotropical region, especially under current conditions of rapid habitat and diversity loss.

Molecular tools can be used to delimitate species and are useful in the study of unexplored groups, serving as first steps for taxonomic description (Kekkonen & Hebert, 2014). Methods such as DNA barcoding can help resolve some of these issues by using molecular markers for species identification (Fišer Pečnikar & Buzan, 2014), but DNA barcode libraries need to be created first. Great effort has been made to build DNA reference libraries in many regions of the world, such as Europe (Weigand et al., 2019), North America (Zhou et al., 2016), and Australia (Carew et al., 2017). In the Neotropics, Costa Rica has developed an initiative to create a DNA barcode database focused on Lepidoptera taxa (Hajibabaei et al., 2006; Janzen & Hallwachs, 2011). However, a recent study in Panama showed that aquatic macroinvertebrate identification using nucleotide databases matches less than 50% of sequences and only to genus and family level (de León et al., 2020). This indicates that more work is needed to strengthen this region's macroinvertebrate knowledge.

Reference databases for the neotropics are limited, thus species delimitation methods are convenient tools for assessing the composition of this understudied biodiversity. Delimitation methods have been established to determine boundaries between species units using sequencing data from DNA barcodes (Fujisawa & Barraclough, 2013; Jones et al., 2011; Ratnasingham & Hebert, 2013; Zhang et al., 2013). The standardized barcode used for metazoans is a short sequence (658 bp) of the mitochondrial gene cytochrome c oxidase subunit I (COI; Coissac et al., 2012; Hebert et al., 2003). Delimitation with single‐locus data allows the assignment of large numbers of operational taxonomic units (OTUs) in short time periods (Puillandre et al., 2012). However, methods that use single‐locus data can also be inconclusive and affect species richness estimates. Therefore, using a combination of methods produces more reliable estimates of species boundaries (Costa‐Silva et al., 2015; Zhou et al., 2019). The most common approaches for single‐locus analyses are the distance‐based and tree‐based methods (Kekkonen et al., 2015). The combination of both methods confirms the species delimitation and helps overcome the constraints of each approach (Blair & Bryson, 2017; Carstens et al., 2013)

Aquatic macroinvertebrate indices have been implemented in the Neotropics using identification to family level. The Biological Monitoring Working Party index, BMWP, first developed for rivers in the United Kingdom (Armitage et al., 1983), has been adapted to the region in Costa Rica (Springer et al., 2010), Colombia (Roldán Pérez, 2003) and Panama (Cornejo et al., 2017). Moreover, in El Salvador, the Hilsenhoff's Family‐level Biotic index (FBI) was adjusted for water quality assessment (Sermeño Chicas et al., 2010). However, Nicaragua has yet to develop its own biological indices and relies on the indices of neighboring countries (Betts et al., 2021). The need to properly evaluate the aquatic diversity in Nicaragua could be accomplished with the implementation of modern DNA‐based strategies (Hering et al., 2018; Leese et al., 2018). Molecular methodologies for aquatic macroinvertebrates delimitation will aid in the development of a standardized biological index for environmental assessment in the country.

The aim of this study was to characterize the macroinvertebrate biodiversity in a stream in Nicaragua, within the eco‐region of dry tropical forest known as the Central American Dry Corridor, using species delimitation methods and biological indices. We used two biological metrics that assigned a score according to their pollution tolerance at family level, the BMWP adapted to Costa Rica, BMWP‐CR (Springer et al., 2010), and the FBI adapted to El Salvador, FBI‐SV‐2010 (Sermeño Chicas et al., 2010). The designation of Operational Taxonomic Units (OTUs) using DNA barcoding was developed using two distance‐based methods, the Assemble Species by Automatic Partitioning method, ASAP (Puillandre et al., 2021), and the Barcode Index Number System, BIN (Ratnasingham & Hebert, 2013), plus two tree‐based methods, the Generalized Mixed Yule Coalescent, GMYC (Fujisawa & Barraclough, 2013) and the Bayesian implementation of the Poisson Tree Process model, PTP (Zhang et al., 2013). This is the first effort in Nicaragua to use molecular delimitation methods as an exploratory tool for understudied aquatic macroinvertebrates in a stream from the Central American Dry Corridor.

## 2 MATERIALS AND METHODS

### 2.1 Study area sites and sample processing

**FIGURE 1:**
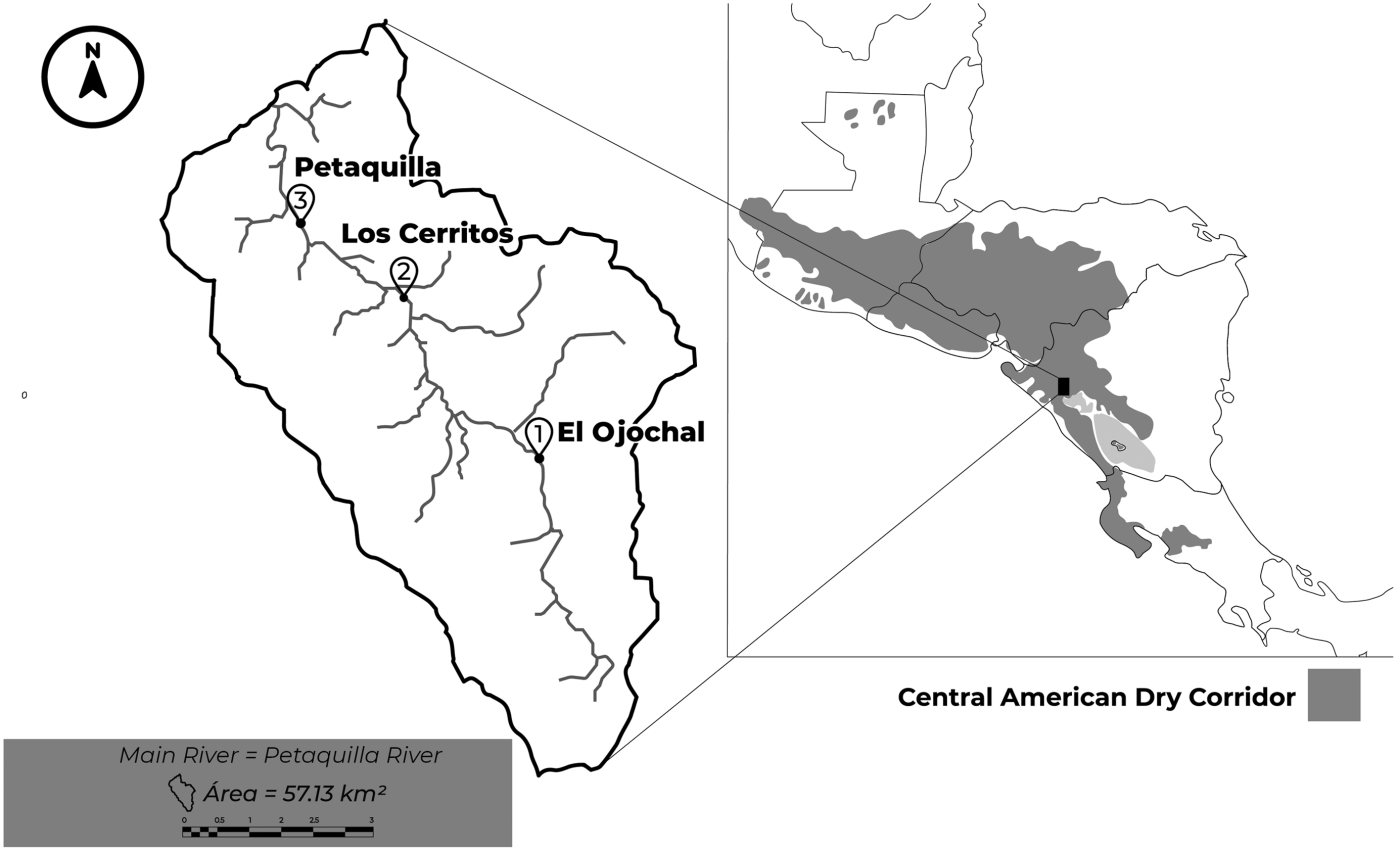
Map showing the location and coordinates of the sampling sites on the Petaquilla River. The gray shadow represents the area covered by the Central American Dry Corridor.

Our study was conducted in the Petaquilla River, located in the Pacific region of Nicaragua, between the 23rd and 29th of May 2019, at the beginning of the rainy season. Samples were collected from three sites located from the headwater to the lower reaches of the river (approx. 6 km). The upper reach, El Ojochal (12°55′05.30″N, 86°27′07.56″W), flows from a waterfall surrounded by a tropical dry forest; the middle reach, Los Cerritos (12°57′02″N, 86°28′27.41″W), located in an agricultural landscape; and the lower reach, Petaquilla (12°57′52.66″N, 86°29′31.22″W), a section of the river closer to small rural communities (Figure 1). Aquatic macroinvertebrates were collected in each site following the described methodology for a multi‐habitat approach according to the Rapid Bioassessment Protocol by EPA (Barbour et al., 1999) and a standardized methodology for rivers in El Salvador (Sermeño Chicas et al., 2010). Each multi‐habitat had a 15‐min sampling effort using a 500‐μm mesh D‐frame dip net. All samples were cleaned up from plant debris in the field and immediately preserved in 95% ethanol. In the laboratory, the ethanol was replaced, first after 48 h, and a second time 72 h later, to better preserve DNA. Specimens were identified to family level using taxonomic keys for neotropical macroinvertebrates (Merritt & Cummins, 1996; Roldán Pérez, 1996). Identification was to family level, complying with standards of regional biological indices (Cárdenas et al., 2007; González‐Alemán et al., 2013; Suarez, 2014).

### 2.2 Biological indices

Water quality assessment was measured using the aquatic macroinvertebrate communities from each sampling site and biological metrics. The Biological Working Party (BMWP) is a score system where each individual family has a value to reflect their pollution tolerance, where pollution‐tolerant families have a high score, and pollution‐intolerant families have lower scores (Armitage et al., 1983). We used the BMWP‐CR adapted to Costa Rica and implemented in their legislation for water protection (Springer et al., 2010). The Hilsenhoff's Family‐level Biotic Index (FBI) is a rapid field‐based assessment that provides average tolerance values of organic pollution to aquatic macroinvertebrates (Hilsenhoff, 1988). We used the adapted index from El Salvador (Sermeño Chicas et al., 2010), FBI‐SV‐2010, which correlates the pollution tolerance values with the relative abundance of each family. The results assign a value between 0 and 10, where closer to zero is for pollution‐intolerant families and higher values are for more tolerant families.

### 2.3 DNA extraction, amplification, and sequencing

Intact individuals from eight taxonomic orders were selected for DNA barcoding analysis (Ephemeroptera, Plecoptera, Trichoptera, Diptera, Hemiptera, Coleoptera, Odonata, Megaloptera). Total genomic DNA was extracted from legs or a piece of body tissue, depending on the specimen, using the DNeasy Blood & Tissue kit (Qiagen), following the manufacturer's instructions. A 658‐bp fragment of the Cytochrome Oxidase I (COI) DNA barcode region was amplified using the universal primers LCO1490 and HCO2198 (Folmer et al., 1994). Polymerase Chain Reactions (PCR) were conducted with a 12.5 μl reaction volume containing 2 μl of DNA template, 3 μl deionized H_2_O, 6.25 μl of Phusion® High‐Fidelity PCR Master Mix with hydrofluoric acid (HF) buffer (New England Biolabs) and 2 μl of each PCR primer (10 μM). The thermocycler conditions were an initial denaturation step at 98°C for 30 s; 30 amplification cycles at 98°C for 10 s, 54°C for 30 s, and 72°C for 35 s; and final extension step at 72°C for 5 min. PCR products were checked by electrophoresis in 1% agarose with an ethidium bromide stain and then were purified using the QIAquick® Nucleotide Removal Kit (Qiagen). Clean DNA was used for cycle sequencing using the forward primer LCO1490 (3.2 μM) and BigDye v3.1 (Thermofisher). This product was sequenced using a SeqStudio genetic analyzer (SeqStudio™, Thermofisher).

### 2.4 Sequences analysis

Sequences were examined using FinchTV 1.4.0 (Geospiza, Inc.; http://www.geospiza.com) to manually edit errors near the beginning and at the end of the sequence. The online version of EMBOSS Transeq (Madeira et al., 2019), was used to perform amino acid translation of the sequences and ensure there were no stop codons present. Then, a multiple sequence alignment was generated for each family using the ClustalW program (Thompson et al., 1994) from MEGA X (Kumar et al., 2018) with default parameters. Phylogenetic trees were constructed using the maximum‐likelihood (ML) analyses according to the estimated best‐fit substitution model (GTR + I + G) in MEGA X. The model function was used to determine the best DNA/Protein model and was selected according to the lowest BIC scores (Bayesian Information Criterion). A total of 1000 nonparametric bootstrap replicates were used (Felsenstein, 1985). Detailed information and sequences for each voucher specimen are available in BOLD Systems under the dataset named “Aquatic Macroinvertebrates from the Nicaragua Dry Corridor Region (DS‐MIANI22)” since 29th April 2022. dx.doi.org/10.5883/DS‐MIANI22.

We used nucleotide databases from BOLD Systems and Genbank to confirm morphological classification to family level of our sequenced specimens. To further identify them to higher taxonomic levels we used the percent identity to reference sequences. For species, we used >97.5% and for genus, 90%–97.4%. Identification to species and genus levels based on the BOLD ID engine was further confirmed using published papers and information from a local museum that reported those species in Nicaragua (http://www.bio‐nica.info/home/index.html). Sequences from the Barcode of Life Data System (BOLD) and NCBI GenBank database were retrieved and included in ML trees to evaluate the accuracy of DNA barcoding in the classification and phylogenetic relationships of species.

### 2.5 Species delimitation

#### 2.5.1 Distance‐based approaches

The Barcode Index Number (BIN) analysis is an online framework integrated into the BOLD System, which consists of a clustering algorithm employing graph theoretic methods to generate operational taxonomic units (OTUs; Ratnasingham & Hebert, 2013). Sequences were automatically assigned to a preexisting BIN in the BOLD database (http://www.boldsystems.org) or as a new BIN on the 21st of October 2021. BIN Discordance analyses were performed to compare the taxonomy of sequences associated with a BIN.

We ran the Assemble Species by Automatic Partitioning (ASAP) analysis (Puillandre et al., 2021) in their web interface (https://bioinfo.mnhn.fr/abi/public/asap/). The ASAP analysis uses pairwise genetic distance to automatically find the barcode gap to divide the dataset into a list of several partitions ranked by a score. This new algorithm overcomes the limitations of its predecessor, ABGD (Puillandre et al., 2012), by selecting the best partition using the “ASAP score” calculated by the probabilities of groups to be panmictic species and the barcode gap widths. The alignment from MEGA X was used as input and the analysis was run using the Jukes‐Cantor model to compute the distances.

#### 2.5.2 Tree‐based approaches

The General Mixed Yule Coalescent (GMYC) model is a quantitative method to delimit OTUs using phylogenetic trees. It is based on a Bayesian inference, fully resolved, ultrametric tree; and delimits species by first using a speciation process and then through coalescent models. The tree was constructed using the BEAST v2.6.6 software package (Bouckaert et al., 2019). The best‐fit model of nucleotide substitution was derived from jModelTest 2.1.10 (Darriba et al., 2012). We selected the HKY + I + G model, according to the Bayesian Information Criteria (BIC). An XML file was created with BEAUti v2.6.6 with the following settings: strict clock model, coalescent constant population prior, and MCMC chain of 40 million sampling every 4000. All other settings were left at default. Tracer v1.7.2 (Rambaut et al., 2018) was used to inspect the effective sample size (ESS) and to determine the burn‐in. The ESS values for all parameters were above 200. A maximum clade credibility tree was summarized in TreeAnnotator v2.6.6 from the output trees constructed in BEAST v2.6.6, using 10% burn‐in and common ancestor height. Figtree v1.4.4 was used to visualize the summarized tree. Single‐threshold GMYC analysis was performed in RStudio (RStudio Team, 2020) using the APE (Paradis & Schliep, 2019) and SPLITS (Ezard et al., 2009) libraries.

We used the Bayesian implementation of the Poisson Tree Process (bPTP) method that can delimit species using nonultrametric phylogenies. The bPTP estimates speciation events in terms of a number of substitutions, therefore it only requires a standard phylogenetic tree as input (Zhang et al., 2013). The maximum‐likelihood (ML) input tree was constructed with RAxML‐NG (Kozlov et al., 2019) on their web server (https://raxml‐ng.vital‐it.ch/) using the GTR + G + I substitution model with 100 nonparametric bootstrap replicates. The bPTP analyses were run on their web server (https://species.h‐its.org/ptp/) for 100,000 MCMC generations, a burn‐in of 0.25, and other parameters as default.

## 3 RESULTS

### 3.1 Biological assessment

A total of 4378 individuals were collected on three sites of Petaquilla river. In El Ojochal, the upper reach, we collected 971 specimens; according to morphological identification, we found 4 phyla, 14 orders, and 43 families. In Los Cerritos, the middle reach, we collected 1411 specimens; according to morphological identification, we found 5 phyla, 13 orders, and 35 families. In Petaquilla, the lower reach, we collected 1996 specimens; according to morphological identification, we found 3 phyla, 13 orders, and 41 families.

We assigned values that correspond to each family following the biological indices metrics to obtain the classification of water quality for each sampling site (Appendix,S1). According to the BMWP‐CR, the three sites correspond to excellent water quality due to their values being >120. However, the values of each site differ between them. In El Ojochal (upper reach), the highest value was 180, followed by Petaquilla (lower reach) with 158, and Los Cerritos (middle reach) with 124. Meanwhile, the Hilsenhoff's FBI‐SV‐2010 designated a different classification at each site. El Ojochal (upper reach) had the best classification among the three sites with possibilities of organic pollution (5.509); Petaquilla (middle reach) had the second‐best classification with possible substantial organic pollution (6.002); and Los Cerritos had the lowest quality levels among the three sites with the possibility of very substantial organic pollution (7.053).

### 3.2 DNA barcoding

Our analyzes focused on barcoding 219 aquatic macroinvertebrates, which amplified successfully. According to morphological identification, our dataset represented 18 families, belonging to 8 different orders (Ephemeroptera [*n* = 36], Diptera [*n* = 41], Hemiptera [*n* = 37], Odonata [*n* = 24], Trichoptera [*n* = 27], Coleoptera [*n* = 27], Megaloptera [*n* = 14], and Plecoptera [*n* = 13]). COI sequence length was >500 bp, after cutting approximately 30 bp at the beginning and 50 bp at the end. The average Phred quality score was >30. DNA sequence translation to amino acid showed that no stop codons were present in the sequences. The mean sequence composition was 16.4% G, 18.8% C, 28.8% A, and 36.1% T, with a mean GC composition of 35.1%. All sequences submitted to the project were successfully uploaded and no issues were reported by BOLD (i.e., sequences with stop codons, contaminated sequences, problematic records flagged), Process IDs: MIANI001‐21‐MIANI219‐21.

The results from the nucleotide BLAST performed using Genbank and BOLD ID engine confirmed the morphological identification to order and family level of our samples. There was an exception with 3 specimens belonging to the family Veliidae that were assigned as Gerridae (Hemiptera order) by both BLAST programs. For the identification to species and genus levels, we used the percent identity and the occurrence reports in Nicaragua to confirm our assignations (Table 1). High percent identity (>97.5%) to reference libraries matched with 27% of our sequences, and most of those sequences belonged to specimens from close geographical regions, especially Costa Rica, Panama, and Mexico. This allowed us to identify 14 species with high confidence. Using percent identities to genus level (97.4%–90.0%), we could identify 39% of our sequences; in total, we identified 33 genera. The Ephemeroptera order had the greatest number of genus and species identified using reference nucleotide databases. In the Trichoptera order, all Philopotamidae specimens were identified to the genus Chimarra.

**TABLE 1:** Total specimens detected using morphological identification and DNA barcoding.

Morphological identification		DNA barcoding			
Order	Number of families	BLAST percent identity to query sequence			Number of sequences
		>97.5%	97.4%–90.0%	<89.9%	
Ephemeroptera	3	8	5	23	36
Diptera	3	16	14	11	41
Hemiptera	3	11	17	9	37
Odonata	3	9	9	6	24
Trichoptera	2	16	0	11	27
Coleoptera	2	0	15	12	27
Megaloptera	1	0	14	0	14
Plecoptera	1	0	13	0	13
Total	18	60 (27.4%)	86 (39.3%)	73 (33.3%)	219

*Note*: Number of families detected per order using morphological identification. Number of sequences matched to query sequences from BOLD database according to percent identity. For species identification, we used >97.5% and for genus, 90%–97.4%. Sequences that had a percent identity <89.9% were left identified to family level.

### 3.3 Phylogenetic trees

We constructed eight phylogenetic trees (Supporting Information), one for each order, using the maximum‐likelihood method. All monophyletic clusters with two or more specimens were associated with high bootstrap values (>99%). The Diptera order had 3 representative families: Tabanidae, Simuliidae, and Chironomidae. The Chironomidae family showed the greatest number of clades among all orders. Some of the Chironomidae clades contained only one specimen. The tree for the order Hemiptera included three families: Notonectidae, Veliidae, and Gerridae. However, the genera *Tachygerris* (Gerridae) and *Rheumatobates* (Gerridae) formed clades with specimens from the Veliidae family. In the tree, the clades composed of the Gerrideae + Veliidae specimens were clustered together within the Veliidae family. Internal splits were found in some monophyletic clusters of the families Simuliidae, Tabanidae, Notonectidae, Gomphidae, Philopotamidae, Corydalidae, and Perlidae (Supporting Information).

To further confirm the identification to genus and species level, we included 39 sequences, downloaded from Genbank and BOLD systems, in the phylogenetic trees of each order (Supporting Information). The analysis showed that specimens identified to genus and species level clustered together with their corresponding reference sequence with a 100% bootstrap value.

### 3.4 Species delimitation methods

#### 3.4.1 Barcode index number (BIN)

The 219 records uploaded to the BOLD System project generated a total of 71 BINs, of which 39 were unique and 32 were nonunique BINs. The orders with the greatest number of unique BINs were the Ephemeroptera (13), Hemiptera (10), and Diptera (8). All previously identified sequences to genus and species using DNA barcoding were assigned to nonunique BINs, supporting their taxonomic classification. Only 2 BINs were reported as discordant, which corresponded to Gerridae specimens, *Tachygerris* sp. and *Rheumatobates* sp., clustered with Veliidae sequences.

#### 3.4.2 Assemble species by automatic partitioning (ASAP)

ASAP analysis generated 10 partitions and ranked them using the lowest ASAP score as the best option. The best ASAP result divided the dataset into 69 groups with a score of 3.5 and a *P*‐value of 6.39e^−4^, followed by the 2nd best partition into 68 groups with a score of 8.0 and a *P*‐value of 8.92e^−4^. Furthermore, a plot of the ASAP score as a function of the threshold distance showed how the partitions with the lowest clustering distance values (d_c_ < 0.01) corresponded to a delimitation of 69 groups.

The Chironomidae family had the greatest number of groups (11) in the best partition (69). In the Hemiptera order, the *Tachygerris* and *Rheumatobates* genera (Gerridae) formed groups with specimens from the Veliidae family. The families Chironomidae, Tabanidae, and Philopotamidae increased in the number of groups as the analysis assigned lower ASAP score.

#### 3.4.3 General mixed yule coalescent (GMYC)

The single‐threshold GMYC model determined 73 ML entities (95% confidence interval = 72–74): 34 ML clusters (95% confidence interval: 33–34) and 39 singletons. The topology of the Bayesian inference tree had some differences compared with the maximum‐likelihood tree, but overall presented the same delimitation of clades.

Diptera was the order with the greatest number of entities (17). It was divided into 11 entities in the Chironomidae, 4 entities in the Tabanidae, and 2 in the Simuliidae family. In the order Hemiptera, both the *Tachygerris* (Gerridae) and *Rheumatobates* (Gerridae) genera formed entities with individuals from the Veliidae family and clustered together in a Veliidae + Gerridae clade in the tree. The Megaloptera and Plecoptera were the orders that had the least number of entities, with a single entity for each.

#### 3.4.4 Bayesian Poisson tree processes (bPTP)

The bPTP delimited a total of 70 OTUs using the maximum‐likelihood solution. The bPTP provided two output trees, maximum‐likelihood, and Bayesian, and both had the same topology as the phylogenetic tree from MEGA X and the ultrametric tree from GMYC. The bPTP delimitation analysis generated fewer splits in some closely related OTUs compared with the GMYC analyses. The Diptera order had a lesser number of OTUs due to the Tabanidae family having only 2 clusters. Moreover, in the bPTP analysis, the Philopotamidae family showed fewer splits, by reducing the number of clusters to five, compared with seven clusters with GMYC.

#### 3.4.5 Methods comparison

**FIGURE 2:**
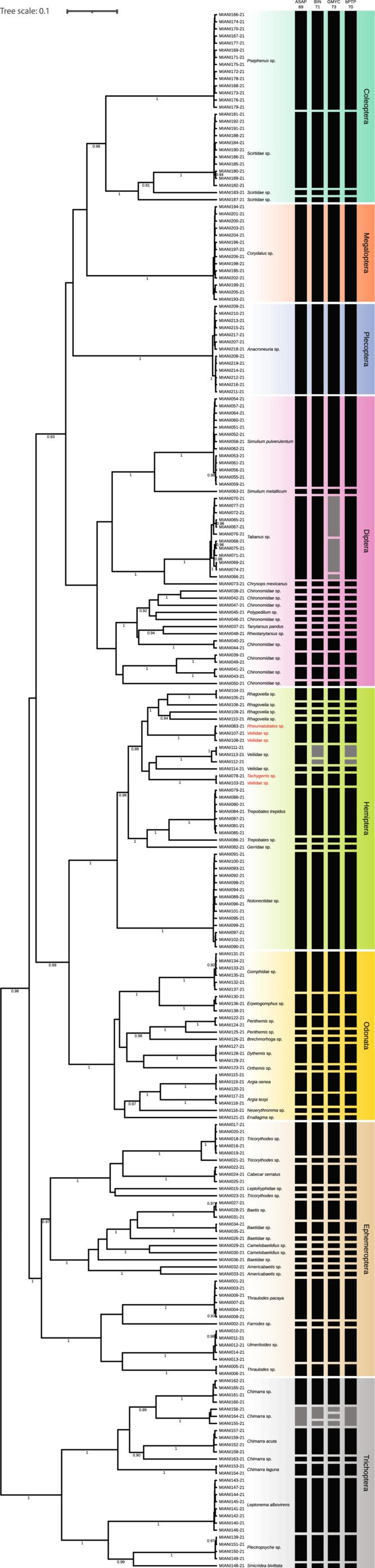
Bayesian inference tree based on COI sequences of aquatic macroinvertebrates with a posteriori probability (BI) showing values higher than 90% on branches. The vertical bars indicate the results of species delimitation methods, Generalized Mixed Yule Coalescent (GMYC), Assemble Species by Automatic Partition (ASAP), Barcode Index Number (BIN), and Bayesian implementation of the Poisson Tree Process (bPTP) separated in taxonomic orders by colors. Gray bars indicate discordant results between the three delimitation methods. Names in red represent discordance from morphological identification.

The count of OTUs in the dataset for each method was: 69 according to ASAP analysis (ASAP score: 3.5, *P*‐value: 6.39e^−4^), 71 for BIN System, 73 for GMYC, and 70 with bPTP analysis (Figure 2; Supporting Information). The comparison of the performance of the four analytical methods (ASAP, BIN, GMYC, bPTP) revealed that 66 OTUs shared the same assignment, which represents 91.8% of the sequences in the dataset. The GMYC count is 73 due to a higher OTUs division in the Tabaniidae and Philopotamidae families, followed by 71 in the BIN system that identified more OTUs in the Veliidae and Philopotamidae family. The bPTP analyses identified 70 OTUs, sharing the same delimitations in the Veliidae family as the BIN method and the same delimitations in the Philopotamidae family with the ASAP analysis. The ASAP analysis had the lowest OTUs count (69) among the four methods.

## 4 DISCUSSION

This is the first study to explore the composition of undocumented macroinvertebrate biodiversity in Nicaragua using traditional taxonomy and molecular delimitation methods. DNA barcode delimitation helped in the characterization of the aquatic macroinvertebrate biodiversity beyond the information provided by morphological classification to family level. We were able to (a) characterize the water quality at each sampling point according to the composition of the aquatic macroinvertebrates communities; (b) identify 18 families using morphological identification and 33 genera and 14 species, using molecular nucleotide databases; (c) divide our dataset into 69–73 OTUs with both distance and tree‐based methods and; (d) obtain a consistent delimitation for 92% of sequences using four delimitation methods. Our results suggest that there are possibly more unidentified species in the studied ecosystem than reported in public nucleotide databases. This delimitation of putative species provided insights into the composition, diversity, and phylogenetic relationships of macroinvertebrates in the dry corridor of Nicaragua.

Biological indices provide information on the macroinvertebrate community response to change (Bonada et al., 2006; Friberg et al., 2011). Using the BMWP‐CR and the FBI‐SV‐2010 indices, we determined that the upper reach of the river, El Ojochal, can be considered a healthy ecosystem with excellent water quality and lower levels of organic pollution compared with the other sites. This section of the river had the highest number of families (43) and many of them included families unique to this site, such as the Ptilodactylidae (Coleoptera), Hydrobiosidae (Trichoptera), and Pseudothelphusidae (Decapoda), which, according to the BMWP‐CR, are families with high requirements of water quality. Also, families such as the Leptophlebiidae (Ephemeroptera), Perlidae (Plecopthera), and Philopotamidae (Trichoptera) had higher FBI‐SV‐2010 scores than in the other sites since this index uses abundance as a factor to classify water quality. Similar water quality and abundance of organisms were found in a study of an area affected by farming but located within a protected area in Nicaragua (Cárdenas et al., 2007). In addition, the presence of Ephemeroptera, Trichoptera, and Plecoptera in El Ojochal is comparable to the diversity found in a study in Bluefields, a zone characterized by the high diversity of species in its rainforest (González‐Alemán et al., 2013). This underscores the importance of conservation efforts in dry forests at the upper reach of rivers from the Dry Corridor region.

The effects of agricultural fields on aquatic communities were reflected in biological indices analysis. The middle reach site, Los Cerritos, had the lowest score for both indices and a low number of families among the three sampling sites. Furthermore, the family Chironomidae (Diptera) in Los Cerritos was the most abundant family, classified as a high tolerance group in the BMWP‐CR index. Other studies in Nicaragua, where rivers showed the same low classification with these indices, also presented high abundance from the Chironomidae family (Rosales, 2012; Suarez, 2014). According to molecular analysis, the Chironomidae family had the greatest number of OTUs, consistently dividing into 11 groups among all four delimitation methods. Using nucleotide databases, we were able to identify a *Tanytarsus pandus* specimen, which is an indicator of sediment pollution in rivers (Carew et al., 2007). The presence of such diverse family groups can be used to elaborate a local index of sensibility to environmental stressors in rivers using both molecular and traditional taxonomy (Lin et al., 2021).

DNA barcodes were compared with nucleotide databases, such as Genbank and BOLD, to further confirm morphological identification to family level, and in some cases, to identify species and genus. All 18 families identified in this study had barcodes deposited in BOLD systems and Genbank databases at least to family level; however, none of them were from Nicaragua. Most families were congruent with their reference sequences, except two specimens from the Veliidae family that were identified as Gerridae. Genus and species delimitation were based on a high identity percentage to reference sequences. Genus reference sequences with >90% identity to barcodes allowed us to identify 33 genera, while species reference sequences with >97.5% identity to barcodes corresponded only to 14 species. Species identification of our specimens matched with reference sequences from Costa Rica, Mexico, Panama, Jamaica, and Venezuela, all countries within the neotropical realm. DNA barcoding has emerged as a useful tool to increase taxonomic resolution in biological assessments (Jackson et al., 2014; Sweeney et al., 2011). As barcode libraries become a widespread practice, the characterization of aquatic macroinvertebrates has the potential to accelerate in Nicaragua.

Molecular analyses using the COI gene revealed the presence of unclear relationships of closely related taxa in the Hemiptera order. Using nucleotide databases, we found three individuals morphologically identified as Veliidae with a high percent identity to reference specimens from the Gerridae family. The ASAP, BIN, GMYC, and bPTP analysis grouped these Veliidae sequences with specimens identified as genera *Rheumatobates* and *Tachygerris* (Gerridae). Previous phylogenetic studies in the Hemiptera order have suggested that the Gerridae and Veliidae families are sister groups based on morphological synapomorphies (Damgaard et al., 2005). Interestingly, in our ML phylogenetic tree, the Gerridae + Veliidae sequences formed clades that clustered together with the Veliidae family. Phylogenetic research has revealed that the Veliidae + Gerridae clade is comprised by several subclades and suggests that these families may be treated as one monophyletic group (Damgaard, 2008). Such ambiguous relationships may lead to morphological misidentification of individuals from the Veliidae and Gerridae groups.

We were able to identify a high diversity of putative species (69–73 OTUs) according to the delimitation methods, even though our dataset consisted of only 219 sequences. Furthermore, BOLD systems identified 39 unique BINs, which represented a considerable percentage (42%) of sequences from our dataset. The limited identification to species level (14) using nucleotide databases compared with OTUs (69–73), from species delimitation methods, indicates that the diversity of aquatic macroinvertebrates in neotropical environments is largely understudied, as suggested by previous studies from Panama (de León et al., 2020; Múrria et al., 2015). Description and identification of species will help to create accurate libraries of neotropical diversity. Species characterization will benefit conservation efforts before neotropical diversity is lost due to anthropogenic impacts such as habitat destruction and contamination (Bowles & Courtney, 2018).

This study was developed in the Central American Dry Corridor, a region that requires special attention since it is vulnerable to climate change and needs to establish effective and multidisciplinary research initiatives to confront its effects (Gotlieb et al., 2019; Kreft et al., 2014). This region is characterized by a dry tropical forest with a high taxonomic richness of aquatic macroinvertebrates, although knowledge of this ecosystem is limited (Kohlmann et al., 2021). This high diversity is derived from the early migratory settlement of dry forest, as this was the first to colonize Central America from Mexico around 2.5 Mya (Willis et al., 2014). Large endemicity of aquatic macroinvertebrates at a small spatial scale has been documented in this type of environment (de León et al., 2020). Spatial isolation, biological function, and habitat variability are all important drivers of macroinvertebrate diversity in the Neotropics (Múrria et al., 2015). However, the dry forest is a heavily threatened tropical ecosystem, which requires restoration efforts with active management to preserve its local endemic species (Griscom & Ashton, 2011).

Insects from the order Ephemeroptera, Plecoptera, and Trichoptera are key components of energy flow in aquatic systems and are used as their own biological indicators (EPT; Barbour et al., 1999; Wallace et al., 1996). In our study, the Ephemeroptera order had the highest number of OTUs (17) and unique BINs (13); however, for each family, we could only identify most of their specimens to genus level using nucleotide databases. Results for the Philopotamidae family (order Trichoptera) were outstanding because we identified only one genus (*Chimarra)*, which was further divided into five different OTUs. This finding is expected because Chimarra is the genus with the largest number of described species in the order and the most abundantly represented in the neotropical region (Kjer et al., 2014). Through our analysis, we were able to identify just one OTU for the Plecoptera order. According to DNA barcoding identification, all specimens belonged to the genus *Anacroneuria*, from the Perlidae family, the only one reported in Nicaragua (Maes, 1998). Despite the use of the Plecoptera order in biological indices, there are not many studies on the ecology and taxonomy of the Perlidae family in Latin America, thus, few species are reported in the region (Alonso‐EguíaLis et al., 2014; Sierra‐Labastidas et al., 2018). Efforts are being made worldwide to develop barcode libraries of insects from the Ephemeroptera, Trichoptera, and Plecoptera orders (Cordero et al., 2017; Gill et al., 2014; Zhou et al., 2010, 2016). The knowledge of these insects' diversity can be used to further improve the biological index assessment of freshwater quality in the Neotropical region.

Delimitation methods using DNA barcode data served as a reliable tool for the fast and objective exploration of the understudied diversity of aquatic macroinvertebrates in the studied region. The comparison between the different approaches helped to confirm the performance of each method (Figure 2). In our study, the four used DNA‐based methods (ASAP, BIN, GMYC, and bPTP) showed a consistent delimitation for 92% of sequences. Even though these methods are commonly used as a first hypothesis for taxonomic assignations, their reliability increases when multiple analyses identify the same species (Blair & Bryson, 2017). Furthermore, such assignations become more relevant in unexplored taxa as those from the dry neotropical region. In our analyses, species delimitation showed congruence with most morphological identification to family level. These results highlight the reliability of the methods used for species delimitation and serve as a proof of concept of the use of DNA barcoding to survey the biodiversity of macroinvertebrates in the studied Neotropical region.

Some limitations of this study include that, while our results show high congruence in species delimitation, certain families presented discrepancies between methods, specifically the Philopotamidae, Veliidae, and Tabanidae families. The Philopotamidae family had three sequences that shared different delimitations with every method. However, this can be explained by the high diversity of species within their clades. Furthermore, studies have found that distance and tree base delimitation methods of single‐locus perform poorly when the number of sample specimens is low (Flot, 2015). Also, the Tabanidae family showed discrepancies only with the GMYC methods, but GMYC has been reported to produce more splits than alternate methods (Kekkonen & Hebert, 2014). Overall, species delimitation methods using a single‐locus are sensitive to effective population size, mutation, and speciation rates, and may also be affected by the species richness in the dataset (Dellicour & Flot, 2018). Considering the limitations of the different methodologies, our results should be considered of preliminary nature and serve as the first hypothesis for the taxonomic exploration of understudied groups. Further studies may be needed, such as additional molecular data and re‐examination of morphological material to clearly interpret species boundaries (Costa‐Silva et al., 2015; Meier et al., 2022; Yeates et al., 2011).

## 5 CONCLUSION

Delimitation methods can serve as tools to describe the diversity, composition, and phylogenetic relationships of aquatic macroinvertebrates in Nicaragua, and to aid in the development of our own biological index. Environmental studies using macroinvertebrates as biological indicators have been used before in Nicaragua to evaluate the effects of human activities such as deforestation and agriculture (Betts et al., 2021). The implementation of molecular approaches can accelerate the speed of exploration in the region and promote the application of modern environmental assessment methodologies based on DNA barcodes. Furthermore, the use of molecular approaches also supports global efforts to incorporate data in the BOLD systems initiative, GenBank, and tree of life projects. Our molecular analyses results showed higher diversity of putative species not perceptible through morphological identification. Therefore, a comprehensive interpretation of molecular and morphological studies must be done to better understand the diversity of macroinvertebrates in the region. This study provides valuable information about the delimitation of putative species and phylogenetic relationships of families that are important biological indicators for the assessment of water quality.

## AUTHOR CONTRIBUTIONS

**Bryant H. Mendoza‐Ramírez:** Formal analysis (supporting); investigation (equal); methodology (lead); writing – original draft (lead); writing – review and editing (equal). **Lucía Páiz‐Medina:** Conceptualization (lead); formal analysis (lead); investigation (equal); writing – original draft (supporting); writing – review and editing (equal). **Thelma Salvatierra‐Suárez:** Investigation (supporting); methodology (supporting). **Nelvia Hernández:** Investigation (supporting); methodology (supporting). **Jorge A. Huete‐Pérez:** Funding acquisition (lead); supervision (lead); writing – review and editing (equal).

## FUNDING INFORMATION

Research was conducted using funds available at the Molecular Biology Center.

## CONFLICT OF INTEREST

The authors declare that they have no competing interests.

### OPEN RESEARCH BADGES

This article has earned Open Data and Open Materials badges. Data and materials are available at http://www.boldsystems.org/index.php/Public_SearchTerms?query=DS‐MIANI22.

## Supporting information


Appendix S1.

Appendix S2.

## Data Availability

DNA sequences of COI mitochondrial gene of delimited specimens were deposited in BOLD SYSTEMS (available at https://doi.org/10.5883/DS‐MIANI22) and cataloged in the Dryad Digital Repository (available at https://doi.org/10.5061/dryad.9cnp5hqnm).
