# Supplementary material for: A survey of aquatic macroinvertebrates in a river from the dry corridor of Nicaragua using biological indices and DNA barcoding
Source: Ecol Evol. 2022 Nov 5;12(11):e9487. doi: 10.1002/ece3.9487 (PMC9636505; doi:10.1002/ece3.9487)
Supplement: Supplementary file 1 — Appendix S1. [file ECE3-12-e9487-s001.docx]

**[Appendices]**

**Appendix 1.** Biological assignment of aquatic macroinvertebrates at the three sampling sites (El Ojochal, Los Cerritos, Petaquilla), according to the BMWP’-CR Index and the IBF-SV-2010 Index. For taxa not found in a sampling site, we used the abbreviation “N/F”.

| **Order** | **Family** | **El Ojochal (Upper reach)** | | **Los Cerritos (Middle reach)** | | **Petaquilla**  **(Lower reach)** | |
| --- | --- | --- | --- | --- | --- | --- | --- |
|  |  | **BMWP’** | **FBI** | **BMWP’** | **FBI** | **BMWP’** | **FBI** |
| **Coleoptera** | **Dryopidae** | 5 | 0.013 | 5 | 0.003 | N/F | N/F |
|  | **Dytiscidae** | 4 | 0.007 | 4 | 0.042 | 4 | 0.008 |
|  | **Elmidae** | 5 | 0.133 | 5 | 0.120 | 5 | 0.272 |
|  | **Psephenidae** | 7 | 0.004 | 7 | 0.009 | 7 | 0.078 |
|  | **Ptilodactylidae** | 7 | 0.006 | N/F | N/F | N/F | N/F |
|  | **Scirtidae** | 4 | 0.106 | 4 | 0.181 | 4 | 0.603 |
|  | **Lampyridae** | N/F | N/F | 4 | 0.002 | N/F | N/F |
|  | **Staphylinidae** | N/F | N/F | 4 | 0.005 | N/F | N/F |
|  | **Hydrophilidae** | N/F | N/F | 3 | 0.042 | 3 | 0.042 |
|  | **Curculionidae** | N/F | N/F | N/F | N/F | 4 | 0.003 |
| **Decapoda** | **Pseudothelphusidae** | 5 | 0.013 | N/F | N/F | N/F | N/F |
| **Diptera** | **Chironomidae** | 2 | 0.192 | 2 | 3.365 | 2 | 0.135 |
|  | **Psychodidae** | 3 | 0.007 | N/F | N/F | N/F | N/F |
|  | **Simuliidae** | 4 | 0.013 | 4 | 0.104 | 4 | 0.003 |
|  | **Stratiomyidae** | 4 | 0.006 | 4 | 0.009 | 4 | 0.212 |
|  | **Tabanidae** | 4 | 0.056 | N/F | N/F | 4 | 0.085 |
|  | **Ceratopogonidae** | 4 | 0.017 | 4 | 0.030 | N/F | N/F |
|  | **Tipulidae** | 4 | 0.026 | N/F | N/F | 4 | 0.003 |
|  | **Muscidae** | N/F | N/F | 4 | 0.007 | N/F | N/F |
| **Ephemeroptera** | **Baetidae** | 5 | 0.244 | 5 | 0.858 | 5 | 0.196 |
|  | **Caenidae** | 4 | 0.022 | 4 | 0.105 | 4 | 0.312 |
|  | **Leptohyphidae** | 5 | 0.882 | 5 | 0.822 | 5 | 1.780 |
|  | **Leptophlebiidae** | 8 | 1.163 | N/F | N/F | 8 | 0.188 |
| **Hemiptera** | **Belostomatidae** | 4 | 0.080 | 4 | 0.005 | 4 | 0.068 |
|  | **Corixidae** | 4 | 0.005 | N/F | N/F | 4 | 0.003 |
|  | **Gerridae** | - | 0.882 | - | 0.515 | - | 0.385 |
|  | **Hydrometridae** | - | 0.000 | N/F | N/F | N/F | N/F |
|  | **Mesoveliidae** | N/F | N/F | N/F | N/F | - | 0.003 |
|  | **Naucoridae** | 4 | 0.031 | 4 | 0.032 | 4 | 0.036 |
|  | **Notonectidae** | 4 | 0.313 | 4 | 0.075 | 4 | 0.065 |
|  | **Veliidae** | - | 0.401 | - | 0.023 | - | 0.065 |
|  | **Gelastocoridae** | N/F | N/F | N/F | N/F | - | 0.005 |
|  | **Nepidae** | N/F | N/F | N/F | N/F | - | 0.005 |
| **Lepidoptera** | **Cramnbidae** | N/F | N/F | N/F | N/F | - | 0.024 |
| **Megaloptera** | **Corydalidae** | 6 | 0.044 | 6 | 0.163 | 6 | 0.046 |
| **Odonata** | **Coenagrionidae** | 4 | 0.366 | 4 | 0.102 | 4 | 0.748 |
|  | **Gomphidae** | 7 | 0.095 | 7 | 0.011 | 7 | 0.072 |
|  | **Libellulidae** | 6 | 0.029 | 6 | 0.079 | 6 | 0.046 |
|  | **Calopterygidae** | N/F | N/F | N/F | N/F | 4 | 0.011 |
|  | **Platystictidae** | 7 | 0.009 | N/F | N/F | 7 | 0.016 |
| **Plecoptera** | **Perlidae** | 9 | 0.048 | N/F | N/F | 9 | 0.002 |
| **Tricoptera** | **Hydrobiosidae** | 9 | 0.004 | N/F | N/F | N/F | N/F |
|  | **Hydropsychidae** | 5 | 0.083 | N/F | N/F | 5 | 0.334 |
|  | **Philopotamidae** | 7 | 0.156 | 7 | 0.038 | 7 | 0.024 |
|  | **Polycentropodidae** | 6 | 0.025 | N/F | N/F | N/F | N/F |
|  | **Hydroptlilidae** | N/F | N/F | 6 | 0.003 | N/F | N/F |
|  | **Calamoceratidae** | N/F | N/F | N/F | N/F | 8 | 0.001 |
| **Trombidiforme** | **Hydrachnidiae** | 4 | 0.000 | 4 | - | 4 | 0.011 |
| **Oligochaeta** | **Tubificidae** | 1 | 0.094 | 1 | 0.158 | 1 | 0.076 |
| **Ancylini** | **Ferrissia** | 3 | 0.000 | N/F | N/F | N/F | N/F |
|  | **Ancylus** | 3 | 0.000 | N/F | N/F | N/F | N/F |
| **Basommatophora** | **Physidae** | N/F | N/F | 3 | 0.135 | 3 | 0.034 |
| **Architaenioglossa** | **Ampullariidae** | 3 | 0.000 | N/F | N/F | N/F | N/F |
| **Tricladida** | **Planariidae** | - | 0.016 | - | 0.008 | N/F | N/F |
| **Nematodo** |  | N/F | N/F | - | 0.004 | N/F | N/F |
| **Index classification** | | **180**  **Excellent Water Quality** | **5.509**  **Organic Pollution** | **124**  **Excellent Water Quality** | **7.053**  **Very Substantial Organic Pollution** | **158 Excellent Water Quality** | **6.002**  **Substantial Organic Pollution** |
