# Supplementary material for: A survey of aquatic macroinvertebrates in a river from the dry corridor of Nicaragua using biological indices and DNA barcoding
Source: Ecol Evol. 2022 Nov 5;12(11):e9487. doi: 10.1002/ece3.9487 (PMC9636505; doi:10.1002/ece3.9487)

**Supporting Information**

For the research article:

**A survey of aquatic macroinvertebrates in a river from the Dry Corridor region of Nicaragua using Biological Indices and DNA Barcoding**

Bryant H. Mendoza-Ramírez^1^, Lucía Páiz-Medina^1^, Thelma Salvatierra-Suárez^1^, Nelvia Hernández^2^, Jorge A. Huete-Pérez^1^

^1^Molecular Biology Center, University of Central America, UCA, Nicaragua. Rotonda R. Dario 150 m Oeste, Managua, Nicaragua

^2^Institute of Interdisciplinary Research in Natural Science, University of Central America, UCA, Nicaragua. Rotonda R. Dario 150 m Oeste, Managua, Nicaragua

**Part I**

**Biological Indices Sampling Scores for the BMWP and IBF indices.**

- **BMWP (Biological Monitoring Working Party)**

**Aquatic Macroinvertebrate score using the adapted version of the BMWP in Costa Rica (BMWP-CR)**

| Score | Family | Order |
| --- | --- | --- |
| 9 | Polythoridae | Odonata |
|  | Heptageniidae | Ephemeroptera |
|  | Perlidae | Plecoptera |
|  | Lepidostomatidae; Odontoceridae; Hydrobiosidae; Ecnomidae | Trichoptera |
|  | Blephariceridae; Athericidae | Diptera |
| 8 | Cordulegastridae; Corduliidae; Aeshnidae; Perilestidae | Odonata |
|  | Leptophlebiidae; | Ephemeroptera |
|  | Glossosomatidae; Limnephilidae; Calamoceratidae; Leptoceridae | Trichoptera |
| 7 | Gomphidae; Lestidae; Megapodagrionidae; Protoneuridae; Platystictidae | Odonata |
|  | Ptilodactylidae; Psephenidae; Lutrochidae | Coleoptera |
|  | Philopotamidae | Trichoptera |
|  | Talitridae; Gammaridae | Crustacea |
| 6 | Libellulidae | Odonata |
|  | Euthyplociidae; Isonychidae | Ephemeroptera |
|  | Hydroptilidae; Polycentropodidae; Xiphocentronidae | Trichoptera |
|  | Corydalidae | Megaloptera |
| 5 | Leptohyphidae; Oligoneuriidae; Polymitarcyidae; Baetidae | Ephemeroptera |
|  | Hydropsychidae; Helicopsychidae | Trichoptera |
|  | Dryopidae; Hydraenidae; Elmidae; Limnichidae | Coleoptera |
|  | Pyralidae | Lepidoptera |
|  | Crustacea | Crustacea |
|  | Turbellaria | Tricladida |
| 4 | Calopterygidae; Coenagrionidae | Odonata |
|  | Caenidae | Ephemeroptera |
|  | Chrysomelidae; Curculionidae; Haliplidae; Lampyridae; Staphylinidae; Dytiscidae; Gyrinidae; Scirtidae; Noteridae | Coleoptera |
|  | Dixidae; Simuliidae; Tipulidae; Dolichopodidae; Empididae; Muscidae; Sciomyzidae; Ceratopogonidae; Stratiomyidae; Tabanidae | Diptera |
|  | Belostomatidae; Corixidae; Naucoridae; Pleidae; Nepidae; Notonectidae | Hemiptera |
|  | Hidracarina | Hidracarina |
| 3 | Hydrophilidae | Coleoptera |
|  | Psychodidae | Diptera |
|  | Asellidae | Crustacea |
|  | Valvatidae; Hydrobiidae; Lymnaeidae; Physidae; Planorbidae; Bithyniidae; Bythinellidae; Sphaeridae | Molusco |
|  | Hirudinea: Glossiphonidae; Hirudidae; Erpobdellidae | Annelida |
| 2 | Chironomidae; Culicidae; Ephydridae | Diptera |
| 1 | Syrphidae | Diptera |
|  | Oligochatea (todas las clases) | Annelida |

**Water quality index of BMWP-CR**

| Score BMWP’–CR | Water Quality | Classification |
| --- | --- | --- |
| >120 | Excellent Water Quality | 1 |
| 101 – 120 | Good Water Quality, not pollutants or stressors | 2 |
| 61 – 100 | Regular Water Quality, moderate pollution | 3 |
| 36 – 60 | Bad Water Quality, polluted | 4 |
| 16 – 35 | Bad Water Quality, very polluted | 5 |
| <15 | Very Bad Water Quality, extremely polluted | 6 |

**Referencies:**

- Springer, M., Ramirez, A. and Hanson, P. (2010) ‘[Fresh water macroinvertebrates of Costa Rica I].’, Revista de biologia tropical, 58 Suppl 4, pp. V–XIII, 1–238.
- MINAE. (2007). Decreto N° 33903. Reglamento para la Evaluación y Clasificación de la Calidad de Cuerpos de Agua Superficiales. Costa Rica: Publicado en La Gaceta N° 178.
- **IBF (Hilsenhoff’s Family-level Biotic Index)**

**Aquatic Macroinvertebrate score using the adapted version of the IBF in El Salvador (IBF-SV-2010)**

| Score | Family | Order |
| --- | --- | --- |
| 0 | Blephariceridae | Diptera |
| 1 | Corduliidae; Platystictidae | Odonata |
|  | Glossosomatidae | Trichoptera |
| 2 | Cordulegasteridae | Odonata |
|  | Perlidae | Plecoptera |
|  | Calamoceratidae; Lepidostomatidae; Odontoceridae Xiphocentronidae | Trichoptera |
| 3 |  | Blattodea |
|  | Gyrinidae; Lampyridae; Ptilodactylidae | Coleoptera |
|  | Heptageniidae | Ephemeroptera |
|  | Polycentropodidae | Trichoptera |
| 4 |  | Bivalvia |
|  | Hydrobiidae | Gastropoda |
|  | Dryopidae; Elmidae; Hydroscaphidae; Noteridae; Psephenidae | Coleoptera |
|  | Pleidae | Hemiptera |
|  | Aeshinidae | Odonata |
|  | Hydrobiosidae; Hydroptilidae; Leptoceridae | Trichoptera |
| 5 |  | Acarina |
|  |  | Nematoda |
|  |  | Planaria |
|  |  | Amphipoda |
|  | Hydraenidae; Limnichidae; Lutrochidae | Coleoptera |
|  |  | Collembola |
|  | Dixidae; Tipulidae | Diptera |
|  | Leptophlebiidae | Ephemeroptera |
|  | Corixidae; Gelastocoridae; Mesoveliidae; Nepidae; Notonectidae; Saldidae; Veliidae | Hemiptera |
|  | Crambidae | Lepidoptera |
|  | Helicopsychidae; Hydropsychidae; Philopotamidae | Trichoptera |
| 6 |  | Decapoda |
|  | Curculionidae; Scirtidae; Staphylinidae | Coleoptera |
|  | Dolichopodidae; Empididae; Simuliidae; Stratiomyidae; Tabanidae | Diptera |
|  | Baetidae; Leptohyphidae | Ephemeroptera |
|  | Gerridae; Hebridae; Naucoridae | Hemiptera |
| 6 | Lestidae | Odonata |
| 7 |  | Hirudinea |
|  | Planorbiidae | Gastropoda |
|  | Dytiscidae; Hydrophilidae | Coleoptera |
|  | Psychodidae | Diptera |
|  | Caenidae | Ephemeroptera |
|  | Belostomatidae; Ochteridae | Hemiptera |
|  | Corydalidae | Megaloptera |
|  | Calopterygidae; Gomphidae; Libellulidae | Odonata |
| 8 | Ceratopogonidae; Chironomidae | Diptera |
| 9 | Physidae | Gastropoda |
|  | Ephydridae; Muscidae | Diptera |
|  | Coenagrionidae | Odonata |
| 10 |  | Oligochaeta |
|  | Culicidae | Diptera |
|  | Syrphidae |  |

**Water Quality indexo f IBF-SV-2010**

| Score  IBF–SV–2010 | Water Quality | Interpretation | Clasificación |
| --- | --- | --- | --- |
| 0.00 – 3.75 | Excellent | Improbable Organic Pollution | 1 |
| 3.76 – 4.25 | Very Good | Probability of Low Organic Pollution | 2 |
| 4.26 – 5.00 | Good | Probability of Medium Organic Pollution | 3 |
| 5.01 – 5.75 | Intermediate | Probability of Organic Pollution | 4 |
| 5.76 – 6.50 | Lower than Intermediate | Probability of Substantial Organic Pollution | 5 |
| 6.51 – 7.25 | Bad | Probability of Very Substantial Organic Pollution | 6 |
| 7.26 – 10.00 | Very Bad | Probability of High Organic Pollution | 7 |

**Referencies:**

- Sermeño Chicas, J. M. et. al. (2010.) ‘Determinación de la calidad ambiental de las aguas de los ríos de El Salvador, utilizando invertebrados acuáticos: índice biológico a nivel de familias de invertebrados acuáticos en El Salvador (IBF-SV-2010). En: Formulación de una guía metodológica estandarizada para determinar la calidad ambiental de las aguas de los ríos de El Salvador, utilizando insectos acuáticos’, Proyecto Universidad de El Salvador (UES) - Organización de los Estados Americanos (OEA), San Salvador: Editorial Universitaria UES, p. 43.

**Part II**

**Phylogenetic trees constructed using Maximum likelihood (ML) analyses in MEGA X**

1. **Coleoptera Order**

**
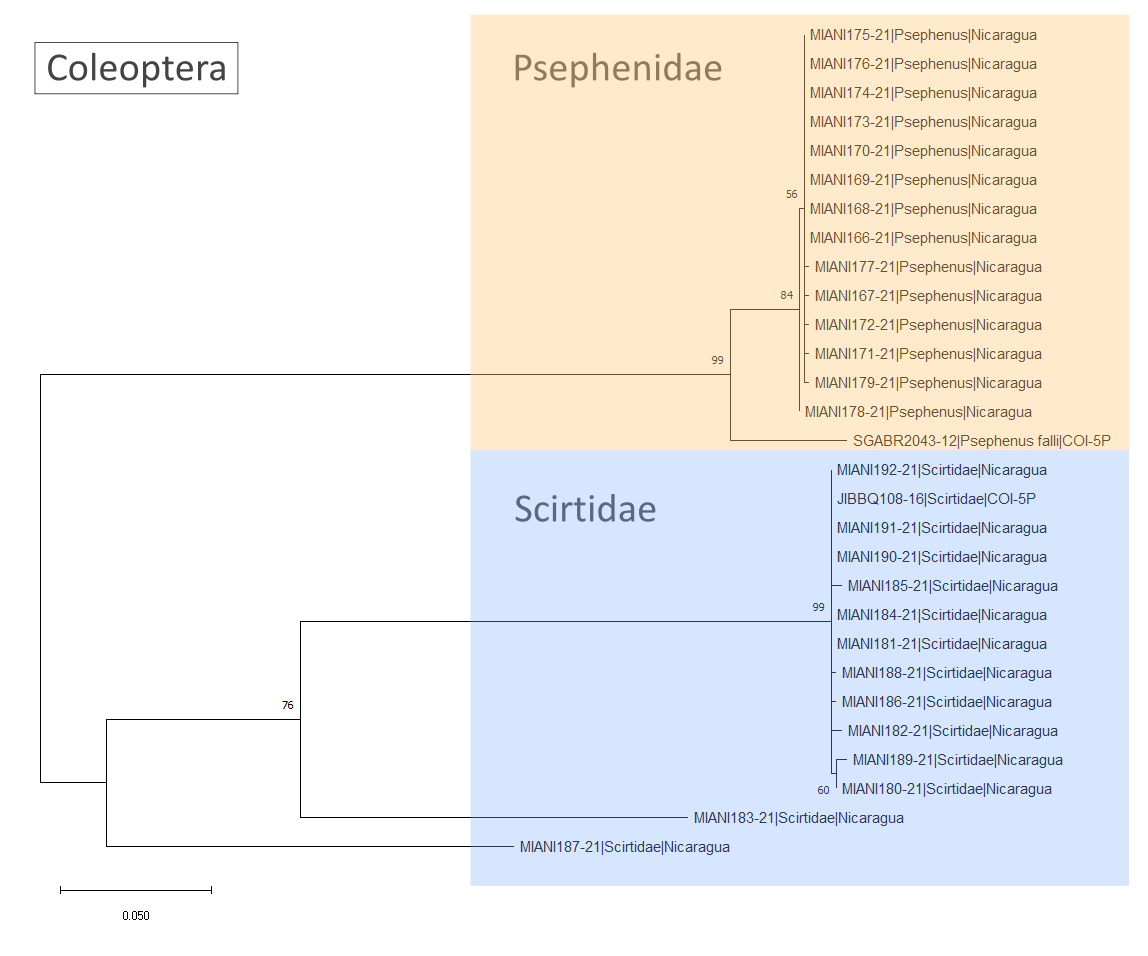
**

1. **Hemiptera Order**

**
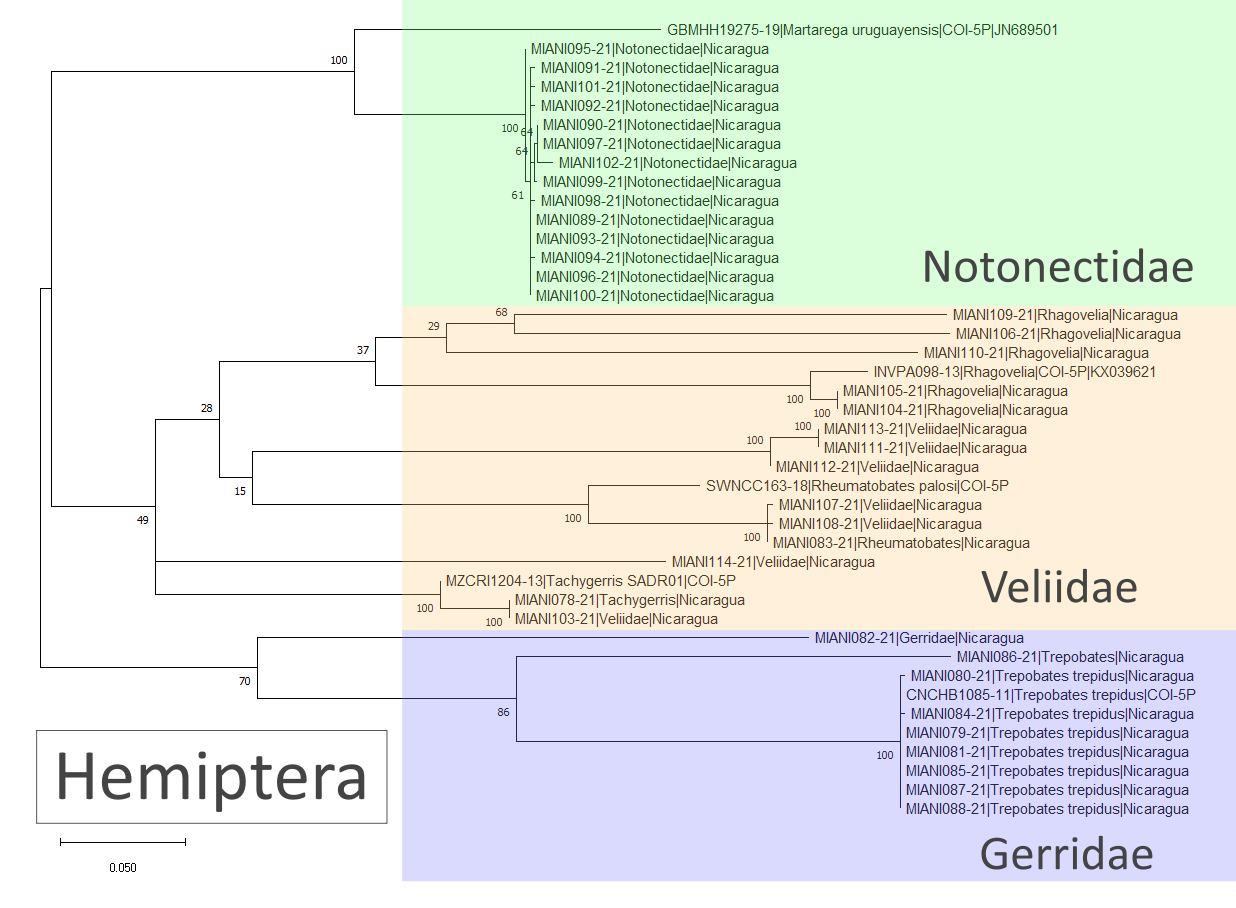

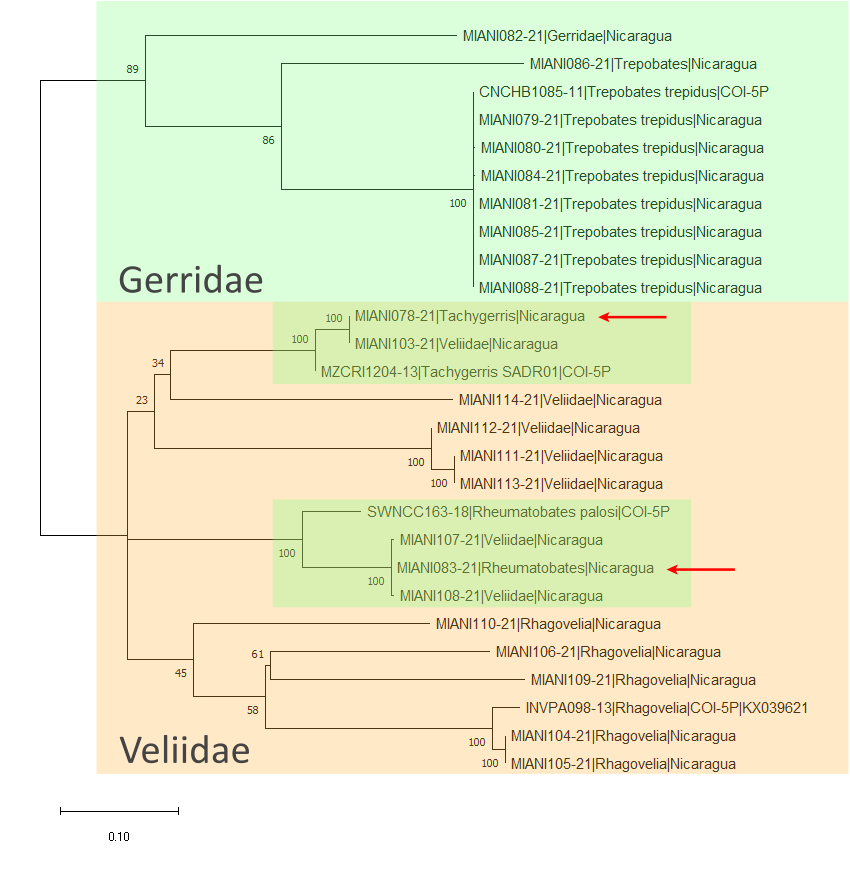
**

1. **Odonata Order**

**
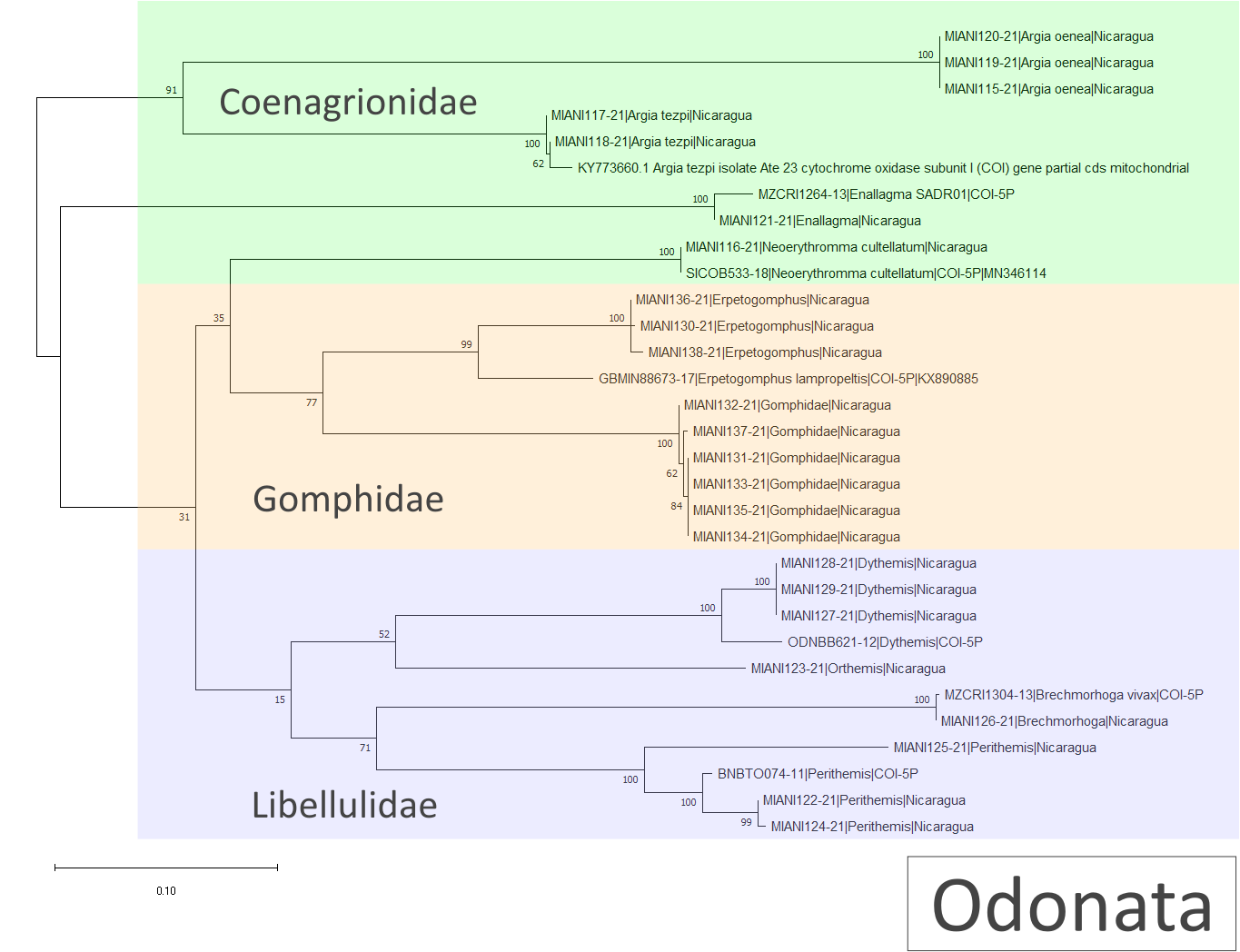
**

1. **Ephemeroptera Order**

**
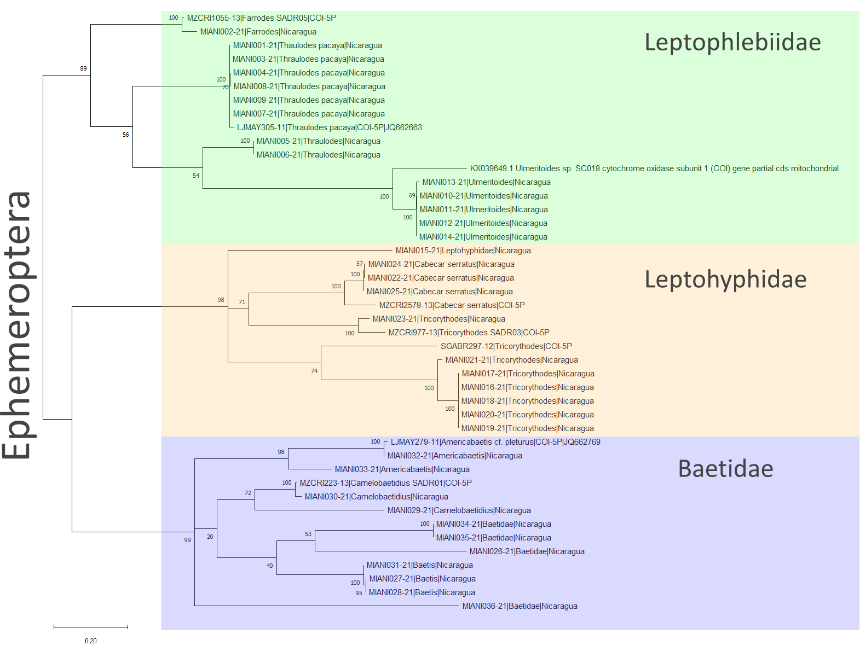
**

1. **Plecopters Order**

**
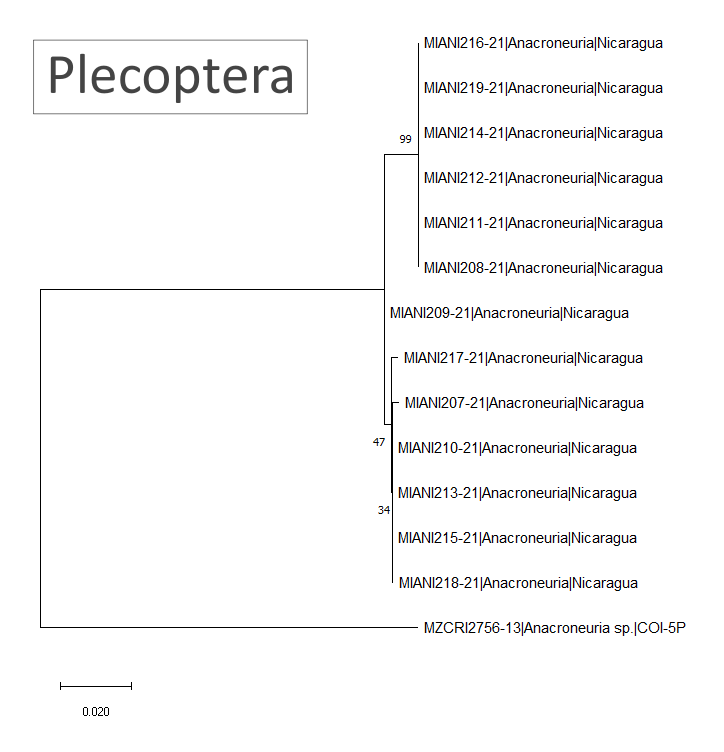
**

1. **Megaloptera Order**

**
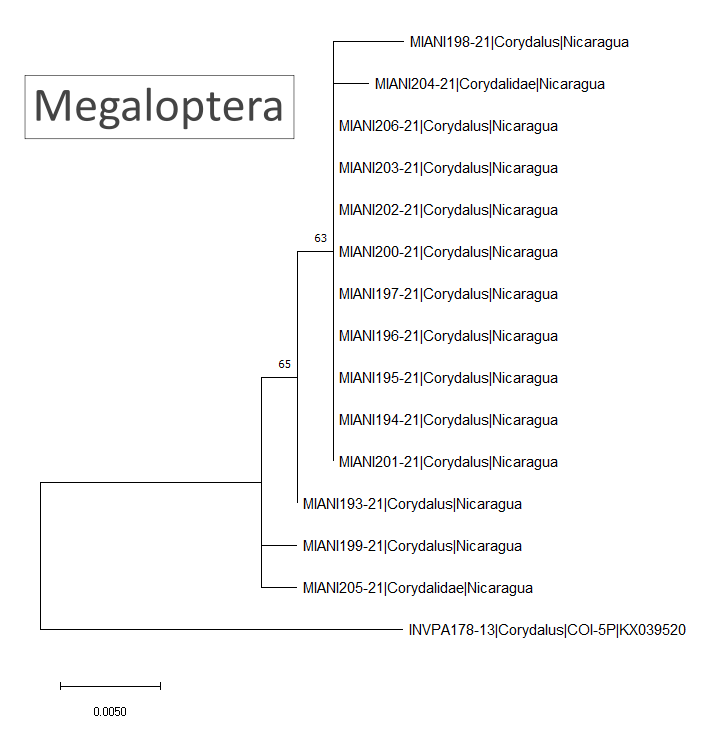
**

1. **Trichoptera Order**

**
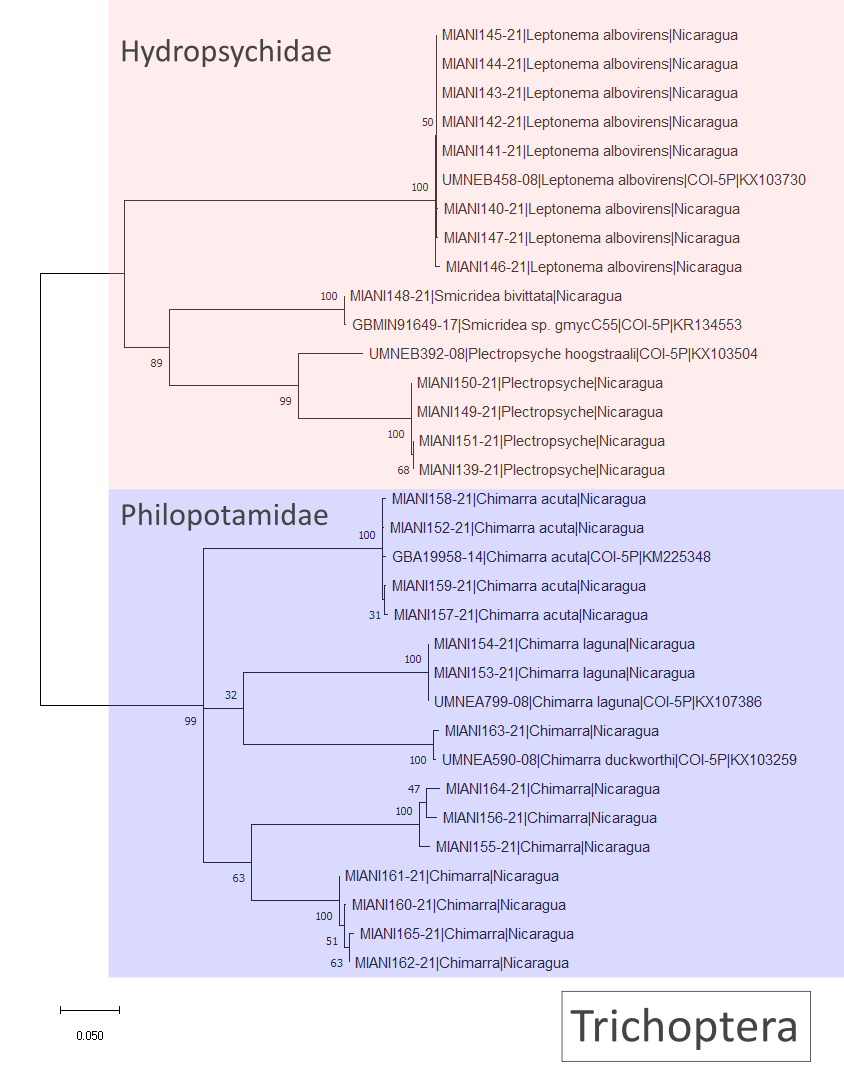
**

1. **Diptera Order**

**
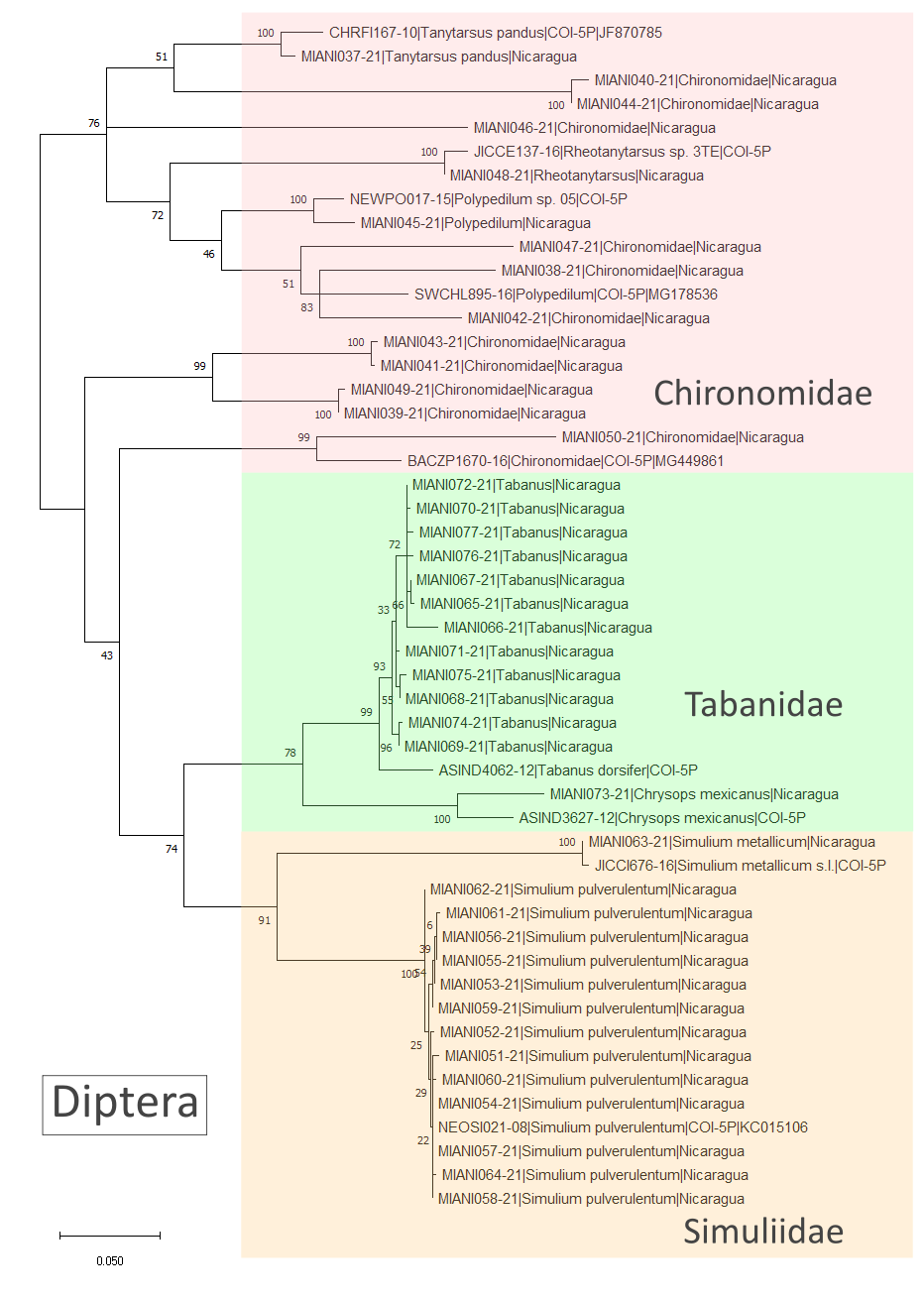
**

**Part III**

**Sequences downloaded from Nucleotide Databases used for ML Phylogenetic Trees**

| Order | Family | ID | Sampling site | Reference |
| --- | --- | --- | --- | --- |
| Ephemeroptera | Leptophlebiidae | LJMAY305-11\|Thraulodes pacaya\|COI-5P\|JQ662663 | Mexico | - |
|  |  | MZCRI1055-13\|Farrodes SADR05\|COI-5P | Costa Rica | - |
|  |  | COMAY016-07\|Ulmeri0074oides acosa\|COI-5P | Costa Rica | - |
|  | Leptohyphidae | MZCRI977-13\|Tricorythodes SADR03\|COI-5P | Costa Rica | - |
|  |  | MZCRI2579-13\|Cabecar serratus\|COI-5P | Costa Rica | - |
|  | Baetidae | MZCRI223-13\|Camelobaetidius SADR01\|COI-5P | Costa Rica | - |
|  |  | LJMAY279-11\|Americabaetis cf. pleturus\|COI-5P\|JQ662769 | Mexico | - |
| Diptera | Chironomidae | CHRFI167-10\|Tanytarsus pandus\|PGD\|MG785645 | Costa Rica | - |
|  |  | NEWPO017-15\|Polypedilum sp. 05\|COI-5P | Brazil | - |
|  |  | JICCE137-16\|Rheotanytarsus sp. 3TE\|COI-5P | Costa Rica | - |
|  |  | SWCHL895-16\|Polypedilum\|COI-5P\|MG178536 | Costa Rica | - |
|  | Simuliidae | NEOSI021-08\|Simulium pulverulentum\|COI-5P\|KC015106 | Costa Rica | - |
|  |  | JICCI676-16\|Simulium metallicum s.l.\|COI-5P | Costa Rica | - |
|  | Tabanidae | ASIND4062-12\|Tabanus dorsifer\|COI-5P | Costa Rica | - |
|  |  | ASIND3627-12\|Chrysops mexicanus\|COI-5P | Costa Rica | - |
| Hemiptera | Gerridae | CNCHB1085-11\|Trepobates trepidus\|COI-5P | Mexico | - |
|  |  | MZCRI1204-13\|Tachygerris SADR01\|COI-5P | Costa Rica | - |
|  |  | SWNCC163-18\|Rheumatobates palosi\|COI-5P | Canada | - |
|  | Notonectidae | GBMHH19275-19\|Martarega uruguayensis\|COI-5P\|JN689501 | Brazil | - |
|  | Veliidae | INVPA098-13\|Rhagovelia\|COI-5P\|KX039621 | Panama | De León et al., 2020 |
| Odonata | Coenagrionidae | SICOB533-18\|Neoerythromma cultellatum\|COI-5P\|MN346114 | Jamaica | - |
|  |  | MZCRI1264-13\|Enallagma SADR01\|COI-5P | Costa Rica | - |
|  |  | KY773660.1 Argia tezpi isolate Ate_23 cytochrome oxidase subunit I (COI) gene | Mexico | Torrés-Pachón et al., 2017 |
|  |  | Argia oenea\|Costa Rica.Puntarenas\|Coenagrionidae\| | Costa Rica | - |
|  | Libellulidae | BNBTO074-11\|Perithemis\|COI-5P | Brazil | - |
|  |  | MZCRI1304-13\|Brechmorhoga vivax\|COI-5P | Costa Rica | - |
|  |  | INVPA122-13\|Libellulidae\|COI-5P\|KX039582 | Panama | De León et al., 2020 |
|  | Gomphidae | INVPA023-13\|Gomphidae\|COI-5P\|KX039556 | Panama | De León,et al 2020 |
|  |  | GBMIN88673-17\|Erpetogomphus lampropeltis\|COI-5P\|KX890885 | - | - |
| Trichoptera | Hydropsychidae | UMNEB458-08\|Leptonema albovirens\|COI-5P\|KX103730 | Costa Rica | Zhou X et al., 2016 |
|  |  | GBMIN91649-17\|Smicridea sp. gmycC55\|COI-5P\|KR134553 | - | - |
|  |  | UMNEB392-08\|Plectropsyche hoogstraali\|COI-5P\|KX103504 | Costa Rica | Zhou X et al., 2016 |
|  | Philopotamidae | UMNEA799-08\|Chimarra laguna\|COI-5P\|KX107386 | Costa Rica | Zhou X et al., 2016 |
|  |  | GBA19958-14\|Chimarra acuta\|COI-5P\|KM225348 | - | Zhou X et al., 2016 |
|  |  | UMNEA590-08\|Chimarra duckworthi\|COI-5P\|KX103259 | Costa Rica | Zhou X et al., 2016 |
| Coleoptera | Psephenidae | SGABR2043-12\|Psephenus falli\|COI-5P | United States | - |
|  | Scirtidae | JIBBQ108-16\|Scirtidae\|COI-5P | Costa Rica | - |
| Megaloptera | Corydalidae | INVPA178-13\|Corydalus\|COI-5P\|KX039520 | Panama | De León et al., 2020 |
| Plecoptera | Perlidae | MZCRI2756-13\|Anacroneuria sp.\|COI-5P | Costa Rica | - |

**References:**

Luis F. De León , Aydeé Cornejo, Ronnie G. Gavilán, Celestino Aguilar Hidden biodiversity in Neotropical streams: DNA barcoding uncovers high endemicity of freshwater macroinvertebrates at small spatial scales PLoS ONE 2020-08-17;15(8): e0231683

Torres-Pachón, M., Novelo-Gutiérrez, R. & Espinosa de los Monteros, A. Phylogenetic analysis of the genus Argia Rambur, 1842 (Odonata: Coenagrionidae), based on morphological characters of larvae and mitochondrial DNA sequences. Org Divers Evol 17, 409–420 (2017). <https://doi.org/10.1007/s13127-017-0325-7>

Zhou X et al. The Trichoptera barcode initiative: a strategy for generating a species-level Tree of Life Philosophocal Transactions of the Royal Society B 2016-08-01;371(1702):25

**Part IV**

**Results of the GMYC analysis. Partitions, Graphs, and histograms of the single-threshold GMYC model 73 ML entities, 34 ML clusters and 39 singletons**

**ML Entities [1] n: 6; id:** MIANI001-21|Thaulodes_pacaya|Nicaragua, MIANI003-21|Thraulodes_pacaya|Nicaragua, MIANI009-21|Thraulodes_pacaya|Nicaragua, MIANI007-21|Thraulodes_pacaya|Nicaragua, MIANI004-21|Thraulodes_pacaya|Nicaragua, MIANI008-21|Thraulodes_pacaya|Nicaragua

**ML Entities [2] n: 2; id:** MIANI005-21|Thraulodes|Nicaragua, MIANI006-21|Thraulodes|Nicaragua

**ML Entities [3] n: 5; id:** MIANI010-21|Ulmeritoides|Nicaragua, MIANI011-21|Ulmeritoides|Nicaragua, MIANI012-21|Ulmeritoides|Nicaragua, MIANI014-21|Ulmeritoides|Nicaragua, MIANI013-21|Ulmeritoides|Nicaragua

**ML Entities [4] n: 5; id:** MIANI016-21|Tricorythodes|Nicaragua, MIANI019-21|Tricorythodes|Nicaragua, MIANI017-21|Tricorythodes|Nicaragua, MIANI020-21|Tricorythodes|Nicaragua, MIANI018-21|Tricorythodes|Nicaragua

**ML Entities [5] n: 3; id:** MIANI022-21|Cabecar_serratus|Nicaragua, MIANI024-21|Cabecar_serratus|Nicaragua, MIANI025-21|Cabecar_serratus|Nicaragua

**ML Entities [6] n: 3; id:** MIANI027-21|Baetis|Nicaragua, MIANI028-21|Baetis|Nicaragua, MIANI031-21|Baetis|Nicaragua

**ML Entities [7] n: 2; id:** MIANI034-21|Baetidae|Nicaragua, MIANI035-21|Baetidae|Nicaragua

**ML Entities [8] n: 2; id:** MIANI040-21|Chironomidae|Nicaragua, MIANI044-21|Chironomidae|Nicaragua

**ML Entities [9] n: 2; id:** MIANI039-21|Chironomidae|Nicaragua, MIANI049-21|Chironomidae|Nicaragua

**ML Entities [10] n: 2; id:** MIANI041-21|Chironomidae|Nicaragua, MIANI043-21|Chironomidae|Nicaragua

**ML Entities [11] n: 13; id:** MIANI051-21|Simulium_pulverulentum|Nicaragua, MIANI054-21|Simulium_pulverulentum|Nicaragua, MIANI057-21|Simulium_pulverulentum|Nicaragua, MIANI064-21|Simulium_pulverulentum|Nicaragua, MIANI060-21|Simulium_pulverulentum|Nicaragua, MIANI052-21|Simulium_pulverulentum|Nicaragua, MIANI058-21|Simulium_pulverulentum|Nicaragua, MIANI062-21|Simulium_pulverulentum|Nicaragua, MIANI053-21|Simulium_pulverulentum|Nicaragua, MIANI061-21|Simulium_pulverulentum|Nicaragua, MIANI056-21|Simulium_pulverulentum|Nicaragua, MIANI055-21|Simulium_pulverulentum|Nicaragua, MIANI059-21|Simulium_pulverulentum|Nicaragua

**ML Entities [12] n: 6; id:** MIANI065-21|Tabanus|Nicaragua, MIANI067-21|Tabanus|Nicaragua, MIANI070-21|Tabanus|Nicaragua, MIANI077-21|Tabanus|Nicaragua, MIANI072-21|Tabanus|Nicaragua, MIANI076-21|Tabanus|Nicaragua

**ML Entities [13] n: 5; id:** MIANI068-21|Tabanus|Nicaragua, MIANI075-21|Tabanus|Nicaragua, MIANI071-21|Tabanus|Nicaragua, MIANI069-21|Tabanus|Nicaragua, MIANI074-21|Tabanus|Nicaragua

**ML Entities [14] n: 14; id:** MIANI166-21|Psephenus|Nicaragua, MIANI174-21|Psephenus|Nicaragua, MIANI170-21|Psephenus|Nicaragua, MIANI167-21|Psephenus|Nicaragua, MIANI177-21|Psephenus|Nicaragua, MIANI169-21|Psephenus|Nicaragua, MIANI171-21|Psephenus|Nicaragua, MIANI175-21|Psephenus|Nicaragua, MIANI172-21|Psephenus|Nicaragua, MIANI178-21|Psephenus|Nicaragua, MIANI168-21|Psephenus|Nicaragua, MIANI173-21|Psephenus|Nicaragua, MIANI176-21|Psephenus|Nicaragua, MIANI179-21|Psephenus|Nicaragua

**ML Entities [15] n: 11; id:** MIANI180-21|Scirtidae|Nicaragua, MIANI189-21|Scirtidae|Nicaragua, MIANI181-21|Scirtidae|Nicaragua, MIANI192-21|Scirtidae|Nicaragua, MIANI191-21|Scirtidae|Nicaragua, MIANI188-21|Scirtidae|Nicaragua, MIANI184-21|Scirtidae|Nicaragua, MIANI190-21|Scirtidae|Nicaragua, MIANI186-21|Scirtidae|Nicaragua, MIANI185-21|Scirtidae|Nicaragua, MIANI182-21|Scirtidae|Nicaragua

**ML Entities [16] n: 14; id:** MIANI193-21|Corydalus|Nicaragua, MIANI199-21|Corydalus|Nicaragua, MIANI205-21|Corydalidae|Nicaragua, MIANI194-21|Corydalus|Nicaragua, MIANI201-21|Corydalus|Nicaragua, MIANI200-21|Corydalus|Nicaragua, MIANI203-21|Corydalus|Nicaragua, MIANI204-21|Corydalidae|Nicaragua, MIANI196-21|Corydalus|Nicaragua, MIANI197-21|Corydalus|Nicaragua, MIANI206-21|Corydalus|Nicaragua, MIANI198-21|Corydalus|Nicaragua, MIANI195-21|Corydalus|Nicaragua, MIANI202-21|Corydalus|Nicaragua

**ML Entities [17] n: 13; id:** MIANI207-21|Anacroneuria|Nicaragua, MIANI218-21|Anacroneuria|Nicaragua, MIANI209-21|Anacroneuria|Nicaragua, MIANI210-21|Anacroneuria|Nicaragua, MIANI213-21|Anacroneuria|Nicaragua, MIANI215-21|Anacroneuria|Nicaragua, MIANI217-21|Anacroneuria|Nicaragua, MIANI208-21|Anacroneuria|Nicaragua, MIANI219-21|Anacroneuria|Nicaragua, MIANI214-21|Anacroneuria|Nicaragua, MIANI211-21|Anacroneuria|Nicaragua, MIANI212-21|Anacroneuria|Nicaragua, MIANI216-21|Anacroneuria|Nicaragua

**ML Entities [18] n: 2; id:** MIANI078-21|Tachygerris|Nicaragua, MIANI103-21|Veliidae|Nicaragua

**ML Entities [19] n: 3; id:** MIANI111-21|Veliidae|Nicaragua, MIANI113-21|Veliidae|Nicaragua, MIANI112-21|Veliidae|Nicaragua

**ML Entities [20] n: 3; id:** MIANI083-21|Rheumatobates|Nicaragua, MIANI107-21|Veliidae|Nicaragua, MIANI108-21|Veliidae|Nicaragua

**ML Entities [21] n: 2; id:** MIANI104-21|Rhagovelia|Nicaragua, MIANI105-21|Rhagovelia|Nicaragua

**ML Entities [22] n: 7; id:** MIANI079-21|Trepobates_trepidus|Nicaragua, MIANI088-21|Trepobates_trepidus|Nicaragua, MIANI080-21|Trepobates_trepidus|Nicaragua, MIANI084-21|Trepobates_trepidus|Nicaragua, MIANI087-21|Trepobates_trepidus|Nicaragua, MIANI081-21|Trepobates_trepidus|Nicaragua, MIANI085-21|Trepobates_trepidus|Nicaragua

**ML Entities [23] n: 14; id:** MIANI089-21|Notonectidae|Nicaragua, MIANI096-21|Notonectidae|Nicaragua, MIANI101-21|Notonectidae|Nicaragua, MIANI091-21|Notonectidae|Nicaragua, MIANI100-21|Notonectidae|Nicaragua, MIANI093-21|Notonectidae|Nicaragua, MIANI092-21|Notonectidae|Nicaragua, MIANI098-21|Notonectidae|Nicaragua, MIANI094-21|Notonectidae|Nicaragua, MIANI095-21|Notonectidae|Nicaragua, MIANI099-21|Notonectidae|Nicaragua, MIANI090-21|Notonectidae|Nicaragua, MIANI097-21|Notonectidae|Nicaragua, MIANI102-21|Notonectidae|Nicaragua

**ML Entities [24] n: 3; id:** MIANI115-21|Argia_oenea|Nicaragua, MIANI119-21|Argia_oenea|Nicaragua, MIANI120-21|Argia_oenea|Nicaragua

**ML Entities [25] n: 2; id:** MIANI117-21|Argia_tezpi|Nicaragua, MIANI118-21|Argia_tezpi|Nicaragua

**ML Entities [26] n: 2; id:** MIANI122-21|Perithemis|Nicaragua, MIANI124-21|Perithemis|Nicaragua

**ML Entities [27] n: 3; id:** MIANI127-21|Dythemis|Nicaragua, MIANI128-21|Dythemis|Nicaragua, MIANI129-21|Dythemis|Nicaragua

**ML Entities [28] n: 3; id:** MIANI130-21|Erpetogomphus|Nicaragua, MIANI136-21|Erpetogomphus|Nicaragua, MIANI138-21|Erpetogomphus|Nicaragua

**ML Entities [29] n: 6; id:** MIANI131-21|Gomphidae|Nicaragua, MIANI134-21|Gomphidae|Nicaragua, MIANI133-21|Gomphidae|Nicaragua, MIANI135-21|Gomphidae|Nicaragua, MIANI132-21|Gomphidae|Nicaragua, MIANI137-21|Gomphidae|Nicaragua

**ML Entities [30] n: 4; id:** MIANI139-21|Plectropsyche|Nicaragua, MIANI151-21|Plectropsyche|Nicaragua, MIANI150-21|Plectropsyche|Nicaragua, MIANI149-21|Plectropsyche|Nicaragua

**ML Entities [31] n: 8; id:** MIANI140-21|Leptonema_albovirens|Nicaragua, MIANI141-21|Leptonema_albovirens|Nicaragua, MIANI142-21|Leptonema_albovirens|Nicaragua, MIANI143-21|Leptonema_albovirens|Nicaragua, MIANI147-21|Leptonema_albovirens|Nicaragua, MIANI144-21|Leptonema_albovirens|Nicaragua, MIANI145-21|Leptonema_albovirens|Nicaragua, MIANI146-21|Leptonema_albovirens|Nicaragua

**ML Entities [32] n: 4; id:** MIANI152-21|Chimarra_acuta|Nicaragua, MIANI157-21|Chimarra_acuta|Nicaragua, MIANI159-21|Chimarra_acuta|Nicaragua, MIANI158-21|Chimarra_acuta|Nicaragua

**ML Entities [33] n: 4; id:** MIANI160-21|Chimarra|Nicaragua, MIANI161-21|Chimarra|Nicaragua, MIANI162-21|Chimarra|Nicaragua, MIANI165-21|Chimarra|Nicaragua

**ML Entities [34] n: 2; id:** MIANI153-21|Chimarra_laguna|Nicaragua, MIANI154-21|Chimarra_laguna|Nicaragua

**ML Entities [35] n: 1; id:** MIANI002-21|Farrodes|Nicaragua

**ML Entities [36] n: 1; id:** MIANI015-21|Leptohyphidae|Nicaragua

**ML Entities [37] n: 1; id:** MIANI023-21|Tricorythodes|Nicaragua

**ML Entities [38] n: 1; id:** MIANI021-21|Tricorythodes|Nicaragua

**ML Entities [39] n: 1; id:** MIANI026-21|Baetidae|Nicaragua

**ML Entities [40] n: 1; id:** MIANI029-21|Camelobaetidius|Nicaragua

**ML Entities [41] n: 1; id:** MIANI030-21|Camelobaetidius|Nicaragua

**ML Entities [42] n: 1; id:** MIANI036-21|Baetidae|Nicaragua

**ML Entities [43] n: 1; id:** MIANI032-21|Americabaetis|Nicaragua

**ML Entities [44] n: 1; id:** MIANI033-21|Americabaetis|Nicaragua

**ML Entities [45] n: 1; id:** MIANI037-21|Tanytarsus_pandus|Nicaragua

**ML Entities [46] n: 1; id:** MIANI048-21|Rheotanytarsus|Nicaragua

**ML Entities [47] n: 1; id:** MIANI038-21|Chironomidae|Nicaragua

**ML Entities [48] n: 1; id:** MIANI042-21|Chironomidae|Nicaragua

**ML Entities [49] n: 1; id:** MIANI047-21|Chironomidae|Nicaragua

**ML Entities [50] n: 1; id:** MIANI045-21|Polypedilum|Nicaragua

**ML Entities [51] n: 1; id:** MIANI046-21|Chironomidae|Nicaragua

**ML Entities [52] n: 1; id:** MIANI050-21|Chironomidae|Nicaragua

**ML Entities [53] n: 1; id:** MIANI063-21|Simulium_metallicum|Nicaragua

**ML Entities [54] n: 1; id:** MIANI066-21|Tabanus|Nicaragua

**ML Entities [55] n: 1; id:** MIANI073-21|Chrysops_mexicanus|Nicaragua

**ML Entities [56] n: 1; id:** MIANI183-21|Scirtidae|Nicaragua

**ML Entities [57] n: 1; id:** MIANI187-21|Scirtidae|Nicaragua

**ML Entities [58] n: 1; id:** MIANI114-21|Veliidae|Nicaragua

**ML Entities [59] n: 1; id:** MIANI106-21|Rhagovelia|Nicaragua

**ML Entities [60] n: 1; id:** MIANI109-21|Rhagovelia|Nicaragua

**ML Entities [61] n: 1; id:** MIANI110-21|Rhagovelia|Nicaragua

**ML Entities [62] n: 1; id:** MIANI086-21|Trepobates|Nicaragua

**ML Entities [63] n: 1; id:** MIANI082-21|Gerridae|Nicaragua

**ML Entities [64] n: 1; id:** MIANI116-21|Neoerythromma_cultellatum|Nicaragua

**ML Entities [65] n: 1; id:** MIANI121-21|Enallagma|Nicaragua

**ML Entities [66] n: 1; id:** MIANI125-21|Perithemis|Nicaragua

**ML Entities [67] n: 1; id:** MIANI126-21|Brechmorhoga|Nicaragua

**ML Entities [68] n: 1; id:** MIANI123-21|Orthemis|Nicaragua

**ML Entities [69] n: 1; id:** MIANI148-21|Smicridea_bivittata|Nicaragua

**ML Entities [70] n: 1; id:** MIANI163-21|Chimarra|Nicaragua

**ML Entities [71] n: 1; id:** MIANI155-21|Chimarra|Nicaragua

**ML Entities [72] n: 1; id:** MIANI156-21|Chimarra|Nicaragua

**ML Entities [73] n: 1; id:** MIANI164-21|Chimarra|Nicaragua


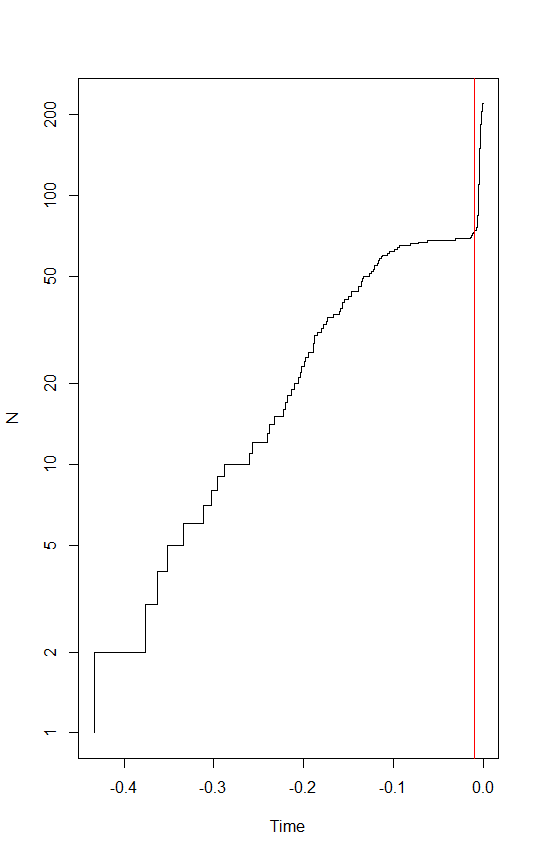

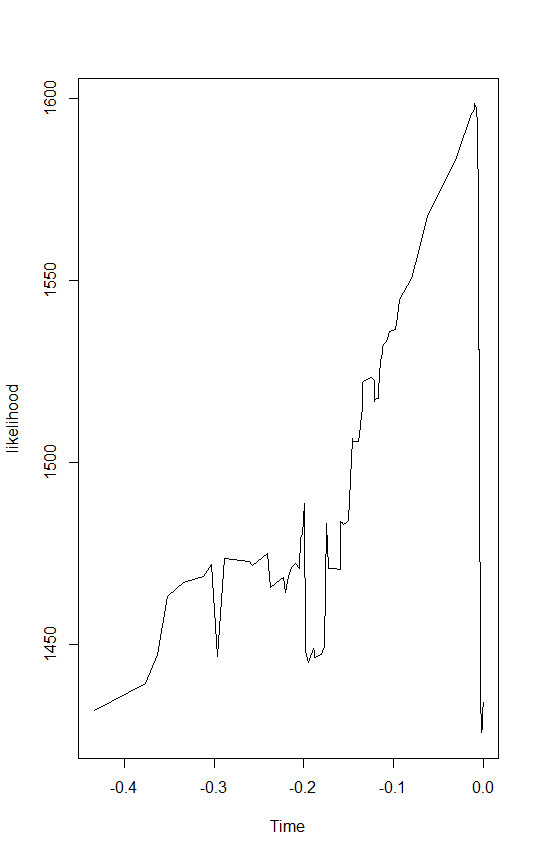


**Results of the ASAP analysis. Best ASAP results: Delimitations, Histogram of distances, Ranked Distances. Best ASAP score 3.50, 69-partition with prior maximal distance P = 6.39e^-04^**

Partition 1

Score: 1

Proba: 6.387696e-04

nb groups:69 (68)

------------------------------------------------------------

**Group[ 1 ] n: 6 ;id:** MIANI001-21|Thaulodes pacaya|Nicaragua MIANI003-21|Thraulodes pacaya|Nicaragua MIANI007-21|Thraulodes pacaya|Nicaragua MIANI004-21|Thraulodes pacaya|Nicaragua MIANI008-21|Thraulodes pacaya|Nicaragua MIANI009-21|Thraulodes pacaya|Nicaragua

**Group[ 2 ] n: 1 ;id:** MIANI002-21|Farrodes|Nicaragua

**Group[ 3 ] n: 2 ;id:** MIANI005-21|Thraulodes|Nicaragua MIANI006-21|Thraulodes|Nicaragua

**Group[ 4 ] n: 5 ;id:** MIANI010-21|Ulmeritoides|Nicaragua MIANI011-21|Ulmeritoides|Nicaragua MIANI012-21|Ulmeritoides|Nicaragua MIANI014-21|Ulmeritoides|Nicaragua MIANI013-21|Ulmeritoides|Nicaragua

**Group[ 5 ] n: 1 ;id:** MIANI015-21|Leptohyphidae|Nicaragua

**Group[ 6 ] n: 5 ;id:** MIANI016-21|Tricorythodes|Nicaragua MIANI017-21|Tricorythodes|Nicaragua MIANI018-21|Tricorythodes|Nicaragua MIANI019-21|Tricorythodes|Nicaragua MIANI020-21|Tricorythodes|Nicaragua

**Group[ 7 ] n: 1 ;id:** MIANI021-21|Tricorythodes|Nicaragua

**Group[ 8 ] n: 3 ;id:** MIANI022-21|Cabecar serratus|Nicaragua MIANI024-21|Cabecar serratus|Nicaragua MIANI025-21|Cabecar serratus|Nicaragua

**Group[ 9 ] n: 1 ;id:** MIANI023-21|Tricorythodes|Nicaragua

**Group[ 10 ] n: 1 ;id:** MIANI026-21|Baetidae|Nicaragua

**Group[ 11 ] n: 3 ;id:** MIANI027-21|Baetis|Nicaragua MIANI028-21|Baetis|Nicaragua MIANI031-21|Baetis|Nicaragua

**Group[ 12 ] n: 1 ;id:** MIANI029-21|Camelobaetidius|Nicaragua

**Group[ 13 ] n: 1 ;id:** MIANI030-21|Camelobaetidius|Nicaragua

**Group[ 14 ] n: 1 ;id:** MIANI032-21|Americabaetis|Nicaragua

**Group[ 15 ] n: 1 ;id:** MIANI033-21|Americabaetis|Nicaragua

**Group[ 16 ] n: 2 ;id:** MIANI034-21|Baetidae|Nicaragua MIANI035-21|Baetidae|Nicaragua

**Group[ 17 ] n: 1 ;id:** MIANI036-21|Baetidae|Nicaragua

**Group[ 18 ] n: 1 ;id:** MIANI037-21|Tanytarsus pandus|Nicaragua

**Group[ 19 ] n: 1 ;id:** MIANI038-21|Chironomidae|Nicaragua

**Group[ 20 ] n: 2 ;id:** MIANI039-21|Chironomidae|Nicaragua MIANI049-21|Chironomidae|Nicaragua

**Group[ 21 ] n: 2 ;id:** MIANI040-21|Chironomidae|Nicaragua MIANI044-21|Chironomidae|Nicaragua

**Group[ 22 ] n: 2 ;id:** MIANI041-21|Chironomidae|Nicaragua MIANI043-21|Chironomidae|Nicaragua

**Group[ 23 ] n: 1 ;id:** MIANI042-21|Chironomidae|Nicaragua

**Group[ 24 ] n: 1 ;id:** MIANI045-21|Polypedilum|Nicaragua

**Group[ 25 ] n: 1 ;id:** MIANI046-21|Chironomidae|Nicaragua

**Group[ 26 ] n: 1 ;id:** MIANI047-21|Chironomidae|Nicaragua

**Group[ 27 ] n: 1 ;id:** MIANI048-21|Rheotanytarsus|Nicaragua

**Group[ 28 ] n: 1 ;id:** MIANI050-21|Chironomidae|Nicaragua

**Group[ 29 ] n: 13 ;id:** MIANI051-21|Simulium pulverulentum|Nicaragua MIANI052-21|Simulium pulverulentum|Nicaragua MIANI054-21|Simulium pulverulentum|Nicaragua MIANI057-21|Simulium pulverulentum|Nicaragua MIANI058-21|Simulium pulverulentum|Nicaragua MIANI060-21|Simulium pulverulentum|Nicaragua MIANI064-21|Simulium pulverulentum|Nicaragua MIANI053-21|Simulium pulverulentum|Nicaragua MIANI055-21|Simulium pulverulentum|Nicaragua MIANI056-21|Simulium pulverulentum|Nicaragua MIANI059-21|Simulium pulverulentum|Nicaragua MIANI061-21|Simulium pulverulentum|Nicaragua MIANI062-21|Simulium pulverulentum|Nicaragua

**Group[ 30 ] n: 1 ;id:** MIANI063-21|Simulium metallicum|Nicaragua

**Group[ 31 ] n: 12 ;id:** MIANI065-21|Tabanus|Nicaragua MIANI067-21|Tabanus|Nicaragua MIANI070-21|Tabanus|Nicaragua MIANI077-21|Tabanus|Nicaragua MIANI072-21|Tabanus|Nicaragua MIANI076-21|Tabanus|Nicaragua MIANI068-21|Tabanus|Nicaragua MIANI071-21|Tabanus|Nicaragua MIANI075-21|Tabanus|Nicaragua MIANI069-21|Tabanus|Nicaragua MIANI074-21|Tabanus|Nicaragua MIANI066-21|Tabanus|Nicaragua

**Group[ 32 ] n: 1 ;id:** MIANI073-21|Chrysops mexicanus|Nicaragua

**Group[ 33 ] n: 2 ;id:** MIANI078-21|Tachygerris|Nicaragua MIANI103-21|Veliidae|Nicaragua

**Group[ 34 ] n: 7 ;id:** MIANI079-21|Trepobates trepidus|Nicaragua MIANI081-21|Trepobates trepidus|Nicaragua MIANI084-21|Trepobates trepidus|Nicaragua MIANI085-21|Trepobates trepidus|Nicaragua MIANI087-21|Trepobates trepidus|Nicaragua MIANI088-21|Trepobates trepidus|Nicaragua MIANI080-21|Trepobates trepidus|Nicaragua

**Group[ 35 ] n: 1 ;id:** MIANI082-21|Gerridae|Nicaragua

**Group[ 36 ] n: 3 ;id:** MIANI083-21|Rheumatobates|Nicaragua MIANI108-21|Veliidae|Nicaragua MIANI107-21|Veliidae|Nicaragua

**Group[ 37 ] n: 1 ;id:** MIANI086-21|Trepobates|Nicaragua

**Group[ 38 ] n: 14 ;id:** MIANI089-21|Notonectidae|Nicaragua MIANI090-21|Notonectidae|Nicaragua MIANI093-21|Notonectidae|Nicaragua MIANI094-21|Notonectidae|Nicaragua MIANI096-21|Notonectidae|Nicaragua MIANI097-21|Notonectidae|Nicaragua MIANI099-21|Notonectidae|Nicaragua MIANI100-21|Notonectidae|Nicaragua MIANI101-21|Notonectidae|Nicaragua MIANI092-21|Notonectidae|Nicaragua MIANI091-21|Notonectidae|Nicaragua MIANI095-21|Notonectidae|Nicaragua MIANI098-21|Notonectidae|Nicaragua MIANI102-21|Notonectidae|Nicaragua

**Group[ 39 ] n: 2 ;id:** MIANI104-21|Rhagovelia|Nicaragua MIANI105-21|Rhagovelia|Nicaragua

**Group[ 40 ] n: 1 ;id:** MIANI106-21|Rhagovelia|Nicaragua

**Group[ 41 ] n: 1 ;id:** MIANI109-21|Rhagovelia|Nicaragua

**Group[ 42 ] n: 1 ;id:** MIANI110-21|Rhagovelia|Nicaragua

**Group[ 43 ] n: 3 ;id:** MIANI111-21|Veliidae|Nicaragua MIANI113-21|Veliidae|Nicaragua MIANI112-21|Veliidae|Nicaragua

**Group[ 44 ] n: 1 ;id:** MIANI114-21|Veliidae|Nicaragua

**Group[ 45 ] n: 3 ;id:** MIANI115-21|Argia oenea|Nicaragua MIANI119-21|Argia oenea|Nicaragua MIANI120-21|Argia oenea|Nicaragua

**Group[ 46 ] n: 1 ;id:** MIANI116-21|Neoerythromma cultellatum|Nicaragua

**Group[ 47 ] n: 2 ;id:** MIANI117-21|Argia tezpi|Nicaragua MIANI118-21|Argia tezpi|Nicaragua

**Group[ 48 ] n: 1 ;id:** MIANI121-21|Enallagma|Nicaragua

**Group[ 49 ] n: 2 ;id:** MIANI122-21|Perithemis|Nicaragua MIANI124-21|Perithemis|Nicaragua

**Group[ 50 ] n: 1 ;id:** MIANI123-21|Orthemis|Nicaragua

**Group[ 51 ] n: 1 ;id:** MIANI125-21|Perithemis|Nicaragua

**Group[ 52 ] n: 1 ;id:** MIANI126-21|Brechmorhoga|Nicaragua

**Group[ 53 ] n: 3 ;id:** MIANI127-21|Dythemis|Nicaragua MIANI128-21|Dythemis|Nicaragua MIANI129-21|Dythemis|Nicaragua

**Group[ 54 ] n: 3 ;id:** MIANI130-21|Erpetogomphus|Nicaragua MIANI136-21|Erpetogomphus|Nicaragua MIANI138-21|Erpetogomphus|Nicaragua

**Group[ 55 ] n: 6 ;id:** MIANI131-21|Gomphidae|Nicaragua MIANI133-21|Gomphidae|Nicaragua MIANI134-21|Gomphidae|Nicaragua MIANI135-21|Gomphidae|Nicaragua MIANI137-21|Gomphidae|Nicaragua MIANI132-21|Gomphidae|Nicaragua

**Group[ 56 ] n: 4 ;id:** MIANI139-21|Plectropsyche|Nicaragua MIANI151-21|Plectropsyche|Nicaragua MIANI149-21|Plectropsyche|Nicaragua MIANI150-21|Plectropsyche|Nicaragua

**Group[ 57 ] n: 8 ;id:** MIANI140-21|Leptonema albovirens|Nicaragua MIANI141-21|Leptonema albovirens|Nicaragua MIANI142-21|Leptonema albovirens|Nicaragua MIANI143-21|Leptonema albovirens|Nicaragua MIANI144-21|Leptonema albovirens|Nicaragua MIANI145-21|Leptonema albovirens|Nicaragua MIANI147-21|Leptonema albovirens|Nicaragua MIANI146-21|Leptonema albovirens|Nicaragua

**Group[ 58 ] n: 1 ;id:** MIANI148-21|Smicridea bivittata|Nicaragua

**Group[ 59 ] n: 4 ;id:** MIANI152-21|Chimarra acuta|Nicaragua MIANI159-21|Chimarra acuta|Nicaragua MIANI158-21|Chimarra acuta|Nicaragua MIANI157-21|Chimarra acuta|Nicaragua

**Group[ 60 ] n: 2 ;id:** MIANI153-21|Chimarra laguna|Nicaragua MIANI154-21|Chimarra laguna|Nicaragua

**Group[ 61 ] n: 3 ;id:** MIANI155-21|Chimarra|Nicaragua MIANI156-21|Chimarra|Nicaragua MIANI164-21|Chimarra|Nicaragua

**Group[ 62 ] n: 4 ;id:** MIANI160-21|Chimarra|Nicaragua MIANI162-21|Chimarra|Nicaragua MIANI165-21|Chimarra|Nicaragua MIANI161-21|Chimarra|Nicaragua

**Group[ 63 ] n: 1 ;id:** MIANI163-21|Chimarra|Nicaragua

**Group[ 64 ] n: 14 ;id:** MIANI166-21|Psephenus|Nicaragua MIANI169-21|Psephenus|Nicaragua MIANI170-21|Psephenus|Nicaragua MIANI168-21|Psephenus|Nicaragua MIANI173-21|Psephenus|Nicaragua MIANI174-21|Psephenus|Nicaragua MIANI175-21|Psephenus|Nicaragua MIANI176-21|Psephenus|Nicaragua MIANI179-21|Psephenus|Nicaragua MIANI177-21|Psephenus|Nicaragua MIANI171-21|Psephenus|Nicaragua MIANI178-21|Psephenus|Nicaragua MIANI167-21|Psephenus|Nicaragua MIANI172-21|Psephenus|Nicaragua

**Group[ 65 ] n: 11 ;id:** MIANI180-21|Scirtidae|Nicaragua MIANI181-21|Scirtidae|Nicaragua MIANI184-21|Scirtidae|Nicaragua MIANI190-21|Scirtidae|Nicaragua MIANI191-21|Scirtidae|Nicaragua MIANI185-21|Scirtidae|Nicaragua MIANI192-21|Scirtidae|Nicaragua MIANI188-21|Scirtidae|Nicaragua MIANI186-21|Scirtidae|Nicaragua MIANI182-21|Scirtidae|Nicaragua MIANI189-21|Scirtidae|Nicaragua

**Group[ 66 ] n: 1 ;id**: MIANI183-21|Scirtidae|Nicaragua

**Group[ 67 ] n: 1 ;id:** MIANI187-21|Scirtidae|Nicaragua

**Group[ 68 ] n: 14 ;id:** MIANI193-21|Corydalus|Nicaragua MIANI194-21|Corydalus|Nicaragua MIANI195-21|Corydalus|Nicaragua MIANI196-21|Corydalus|Nicaragua MIANI197-21|Corydalus|Nicaragua MIANI200-21|Corydalus|Nicaragua MIANI201-21|Corydalus|Nicaragua MIANI202-21|Corydalus|Nicaragua MIANI203-21|Corydalus|Nicaragua MIANI206-21|Corydalus|Nicaragua MIANI204-21|Corydalidae|Nicaragua MIANI198-21|Corydalus|Nicaragua MIANI199-21|Corydalus|Nicaragua MIANI205-21|Corydalidae|Nicaragua

**Group[ 69 ] n: 13 ;id:** MIANI207-21|Anacroneuria|Nicaragua MIANI210-21|Anacroneuria|Nicaragua MIANI213-21|Anacroneuria|Nicaragua MIANI215-21|Anacroneuria|Nicaragua MIANI218-21|Anacroneuria|Nicaragua MIANI209-21|Anacroneuria|Nicaragua MIANI217-21|Anacroneuria|Nicaragua MIANI208-21|Anacroneuria|Nicaragua MIANI211-21|Anacroneuria|Nicaragua MIANI212-21|Anacroneuria|Nicaragua MIANI214-21|Anacroneuria|Nicaragua MIANI216-21|Anacroneuria|Nicaragua MIANI219-21|Anacroneuria|Nicaragua


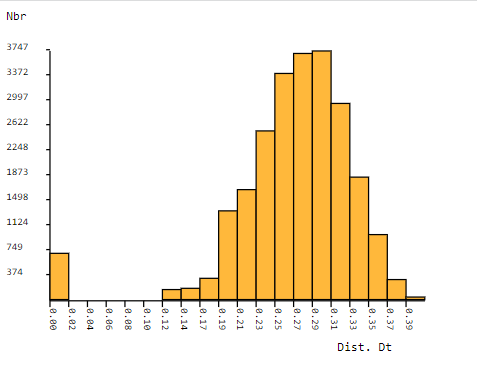


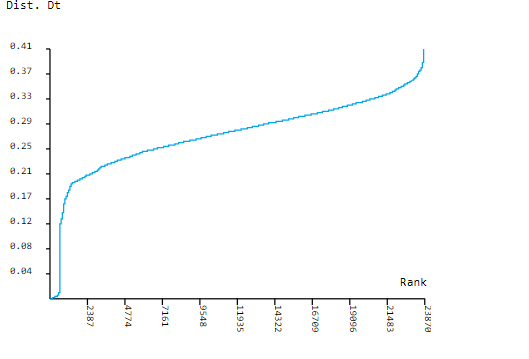


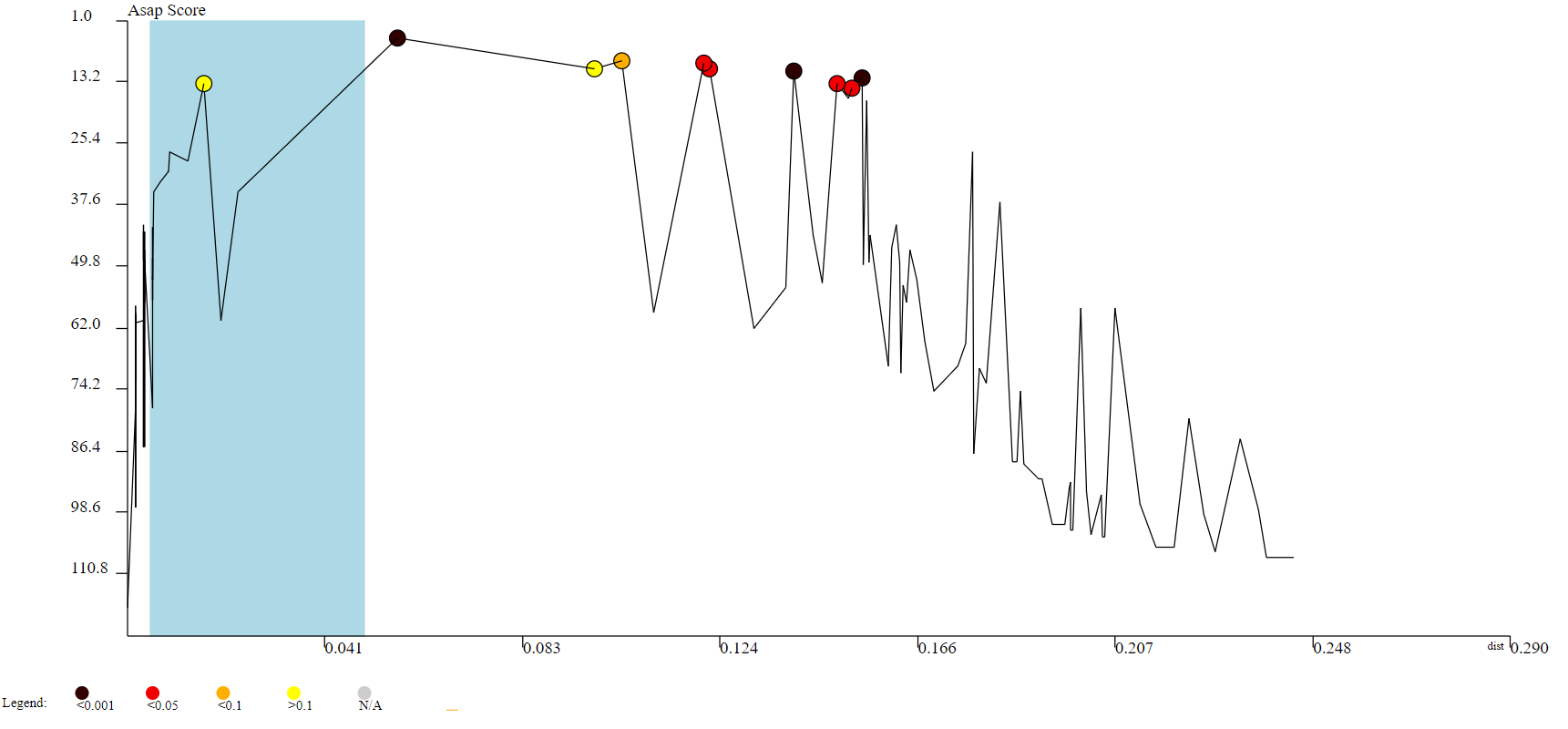


**Results of the BIN analysis. 71 BINS generated by BOLD SYSTEM. 39 Unique BINs. 32 Non Unique BINs.**

Unique Bins (39) – Members 92

**BOLD:AEK9051, n: 1 ;id:** MIANI021-21

**BOLD:AEL5171, n: 1 ;id:** MIANI109-21

**BOLD:AEK7546, n:1 ;id:** MIANI086-21

**BOLD:AEK8202, n:1 ;id:** MIANI050-21

**BOLD:AEL4079, n:1 ;id:** MIANI110-21

**BOLD:AEK4575, n:1 ;id:** MIANI042-21

**BOLD:AEK9049, n:1 ;id:** MIANI023-21

**BOLD:AEK9487, n:1 ;id:** MIANI048-21

**BOLD:AEK7318, n:1 ;id:** MIANI045-21

**BOLD:AEK6191, n:1 ;id:** MIANI187-21

**BOLD:AEL1377, n:1 ;id:** MIANI082-21

**BOLD:AEK7719, n:1 ;id:** MIANI002-21

**BOLD:AEK8192, n:1 ;id:** MIANI029-21

**BOLD:AEL0793, n:1 ;id:** MIANI015-21

**BOLD:AEL4679, n:1 ;id:** MIANI155-21

**BOLD:AEK8490, n:1 ;id:** MIANI073-21

**BOLD:AEL2884, n:1 ;id:** MIANI046-21

**BOLD:AEL6839, n:1 ;id:** MIANI106-21

**BOLD:AEL3517, n:1 ;id:** MIANI047-21

**BOLD:AEL6584, n:1 ;id:** MIANI033-21

**BOLD:AEK4472, n:1 ;id:** MIANI038-21

**BOLD:AEK9388, n:1 ;id:** MIANI114-21

**BOLD:AEK6294, n:1 ;id:** MIANI036-21

**BOLD:AEK9050, n:5 ;id:** MIANI016-21,MIANI017-21,MIANI018-21,MIANI019-21,MIANI020-21

**BOLD:AEL1208, n:2 ;id:** MIANI111-21,MIANI113-21

**BOLD:AEL5078, n:14 ;id:** MIANI166-21,MIANI167-21,MIANI168-21,MIANI169-21,MIANI170-21,MIANI171-21,MIANI172-21,MIANI173-21,MIANI174-21,MIANI175-21,MIANI176-21,MIANI177-21,MIANI178-21,MIANI179-21

**BOLD:AEL5180, n:5 ;id:** MIANI010-21,MIANI011-21,MIANI012-21,MIANI013-21,MIANI014-21

**BOLD:AEL1157, n:3 ;id:** MIANI022-21,MIANI024-21,MIANI025-21

**BOLD:AEL2611, n:2 ;id:** MIANI034-21,MIANI035-21

**BOLD:AEK9488, n:2 ;id:** MIANI122-21,MIANI124-21

**BOLD:AEK5891, n:4 ;id:** MIANI139-21,MIANI149-21,MIANI150-21,MIANI151-21

**BOLD:AEK9702, n:3 ;id:** MIANI130-21,MIANI136-21,MIANI138-21

**BOLD:AEK9509, n:3 ;id:** MIANI027-21,MIANI028-21,MIANI031-21

**BOLD:AEL6840, n:2 ;id:** MIANI104-21,MIANI105-21

**BOLD:AEL0032, n:13 ;id:** MIANI207-21,MIANI208-21,MIANI209-21,MIANI210-21,MIANI211-21,MIANI212-21,MIANI213-21,MIANI214-21,MIANI215-21,MIANI216-21,MIANI217-21,MIANI218-21,MIANI219-21

**BOLD:AEL4680, n:4 ;id:** MIANI160-21,MIANI161-21,MIANI162-21,MIANI165-21

**BOLD:AEL0630, n:2 ;id:** MIANI005-21,MIANI006-21

**BOLD:AEK8315, n:2 ;id:** MIANI078-21,MIANI103-21

**BOLD:AEK4584, n:3 ;id:** MIANI083-21,MIANI107-21,MIANI108-21

Non Unique Bins (32) – Members 127

**BOLD:ACF9712, n:1 ;id:** MIANI030-21

**BOLD:ADS8708, n:1 ;id:** MIANI123-21

**BOLD:AAB3269, n:1 ;id:** MIANI063-21

**BOLD:ADQ7026, n:1 ;id:** MIANI116-21

**BOLD:AAZ4664, n:1 ;id:** MIANI032-21

**BOLD:ADA4146, n:1 ;id:** MIANI112-21

**BOLD:ACG0723, n:1 ;id:** MIANI126-21

**BOLD:AEF8163, n:1 ;id:** MIANI026-21

**BOLD:AAJ0479, n:1 ;id:** MIANI163-21

**BOLD:AEL3529, n:1 ;id:** MIANI037-21

**BOLD:ADA7330, n:1 ;id:** MIANI183-21

**BOLD:ACG0658, n:1 ;id:** MIANI121-21

**BOLD:AAD7770, n:1 ;id:** MIANI148-21

**BOLD:AAG7267, n:1 ;id:** MIANI125-21

**BOLD:ACQ0111, n:4 ;id:** MIANI152-21,MIANI157-21,MIANI158-21,MIANI159-21

**BOLD:ADA4457, n:3 ;id:** MIANI127-21,MIANI128-21,MIANI129-21

**BOLD:ADA5170, n:6 ;id:** MIANI131-21,MIANI132-21,MIANI133-21,MIANI134-21,MIANI135-21,MIANI137-21

**BOLD:ADL7462, n:2 ;id:** MIANI040-21,MIANI044-21

**BOLD:ABW1707, n:7 ;id:** MIANI079-21,MIANI080-21,MIANI081-21,MIANI084-21,MIANI085-21,MIANI087-21,MIANI088-21

**BOLD:ADB3967, n:11 ;id:** MIANI180-21,MIANI181-21,MIANI182-21,MIANI184-21,MIANI185-21,MIANI186-21,MIANI188-21,MIANI189-21,MIANI190-21,MIANI191-21,MIANI192-21

**BOLD:ABW2531, n:12 ;id:** MIANI065-21,MIANI066-21,MIANI067-21,MIANI068-21,MIANI069-21,MIANI070-21,MIANI071-21,MIANI072-21,MIANI074-21,MIANI075-21,MIANI076-21,MIANI077-21

**BOLD:AEL4678, n:2 ;id:** MIANI156-21,MIANI164-21

**BOLD:AAC4696, n:13 ;id:** MIANI051-21,MIANI052-21,MIANI053-21,MIANI054-21,MIANI055-21,MIANI056-21,MIANI057-21,MIANI058-21,MIANI059-21,MIANI060-21,MIANI061-21,MIANI062-21,MIANI064-21

**BOLD:AAB8597, n:8 ;id:** MIANI140-21,MIANI141-21,MIANI142-21,MIANI143-21,MIANI144-21,MIANI145-21,MIANI146-21,MIANI147-21

**BOLD:AEJ5794, n:3 ;id:** MIANI115-21,MIANI119-21,MIANI120-21

**BOLD:ACP0869, n:14 ;id:** MIANI193-21,MIANI194-21,MIANI195-21,MIANI196-21,MIANI197-21,MIANI198-21,MIANI199-21,MIANI200-21,MIANI201-21,MIANI202-21,MIANI203-21,MIANI204-21,MIANI205-21,MIANI206-21

**BOLD:AAZ4759, n:6 ;id:** MIANI001-21,MIANI003-21,MIANI004-21,MIANI007-21,MIANI008-21,MIANI009-21

**BOLD:ACD4877, n:2 ;id:** MIANI039-21,MIANI049-21

**BOLD:AAW6402, n:2 ;id:** MIANI153-21,MIANI154-21

**BOLD:AEL3438, n:2 ;id:** MIANI117-21,MIANI118-21

**BOLD:ACK0293, n:2 ;id:** MIANI041-21,MIANI043-21

**BOLD:ADN3958, n:14 ;id:** MIANI089-21,MIANI090-21,MIANI091-21,MIANI092-21,MIANI093-21,MIANI094-21,MIANI095-21,MIANI096-21,MIANI097-21,MIANI098-21,MIANI099-21,MIANI100-21,MIANI101-21,MIANI102-21

**Results of the bPTP analysis. Maximum Likelihood Solution, MCMC analysis convergence, Delimitation of 70 species Best Results**

# Max likilhood partition

**Species 1 (support = 1.000)**

MIANI036-21|Baetidae|Nicaragua

**Species 2 (support = 0.904)**

MIANI179-21|Psephenus|Nicaragua,MIANI174-21|Psephenus|Nicaragua, MIANI177-21|Psephenus|Nicaragua,MIANI178-21|Psephenus|Nicaragua, MIANI173-21|Psephenus|Nicaragua,MIANI167-21|Psephenus|Nicaragua, MIANI175-21|Psephenus|Nicaragua,MIANI172-21|Psephenus|Nicaragua, MIANI166-21|Psephenus|Nicaragua,MIANI169-21|Psephenus|Nicaragua, MIANI170-21|Psephenus|Nicaragua,MIANI171-21|Psephenus|Nicaragua, MIANI176-21|Psephenus|Nicaragua,MIANI168-21|Psephenus|Nicaragua

**Species 3 (support = 0.917)**

MIANI199-21|Corydalus|Nicaragua,MIANI205-21|Corydalidae|Nicaragua, MIANI193-21|Corydalus|Nicaragua,MIANI196-21|Corydalus|Nicaragua, MIANI202-21|Corydalus|Nicaragua,MIANI195-21|Corydalus|Nicaragua, MIANI201-21|Corydalus|Nicaragua,MIANI197-21|Corydalus|Nicaragua, MIANI194-21|Corydalus|Nicaragua,MIANI206-21|Corydalus|Nicaragua, MIANI200-21|Corydalus|Nicaragua,MIANI198-21|Corydalus|Nicaragua, MIANI204-21|Corydalidae|Nicaragua,MIANI203-21|Corydalus|Nicaragua

**Species 4 (support = 0.909)**

MIANI207-21|Anacroneuria|Nicaragua,MIANI215-21|Anacroneuria|Nicaragua, MIANI218-21|Anacroneuria|Nicaragua,MIANI217-21|Anacroneuria|Nicaragua, MIANI216-21|Anacroneuria|Nicaragua,MIANI214-21|Anacroneuria|Nicaragua, MIANI211-21|Anacroneuria|Nicaragua,MIANI219-21|Anacroneuria|Nicaragua, MIANI212-21|Anacroneuria|Nicaragua,MIANI208-21|Anacroneuria|Nicaragua, MIANI209-21|Anacroneuria|Nicaragua,MIANI210-21|Anacroneuria|Nicaragua, MIANI213-21|Anacroneuria|Nicaragua

**Species 5 (support = 1.000)**

MIANI148-21|Smicridea bivittata|Nicaragua

**Species 6 (support = 0.899)**

MIANI139-21|Plectropsyche|Nicaragua,MIANI151-21|Plectropsyche|Nicaragua, MIANI149-21|Plectropsyche|Nicaragua,MIANI150-21|Plectropsyche|Nicaragua

**Species 7 (support = 0.965)**

MIANI141-21|Leptonema albovirens|Nicaragua,MIANI147-21|Leptonema albovirens|Nicaragua, MIANI144-21|Leptonema albovirens|Nicaragua,MIANI146-21|Leptonema albovirens|Nicaragua, MIANI143-21|Leptonema albovirens|Nicaragua,MIANI145-21|Leptonema albovirens|Nicaragua, MIANI142-21|Leptonema albovirens|Nicaragua,MIANI140-21|Leptonema albovirens|Nicaragua

**Species 8 (support = 1.000)**

MIANI045-21|Polypedilum|Nicaragua

**Species 9 (support = 1.000)**

MIANI046-21|Chironomidae|Nicaragua

**Species 10 (support = 0.827)**

MIANI040-21|Chironomidae|Nicaragua,MIANI044-21|Chironomidae|Nicaragua

**Species 11 (support = 1.000)**

MIANI050-21|Chironomidae|Nicaragua

**Species 12 (support = 1.000)**

MIANI163-21|Chimarra|Nicaragua

**Species 13 (support = 0.917)**

MIANI162-21|Chimarra|Nicaragua,MIANI160-21|Chimarra|Nicaragua,

MIANI165-21|Chimarra|Nicaragua,MIANI161-21|Chimarra|Nicaragua

**Species 14 (support = 0.522)**

MIANI153-21|Chimarra laguna|Nicaragua,MIANI154-21|Chimarra laguna|Nicaragua

**Species 15 (support = 1.000)**

MIANI015-21|Leptohyphidae|Nicaragua

**Species 16 (support = 1.000)**

MIANI023-21|Tricorythodes|Nicaragua

**Species 17 (support = 1.000)**

MIANI114-21|Veliidae|Nicaragua

**Species 18 (support = 0.493)**

MIANI078-21|Tachygerris|Nicaragua,MIANI103-21|Veliidae|Nicaragua

**Species 19 (support = 0.630)**

MIANI095-21|Notonectidae|Nicaragua,MIANI090-21|Notonectidae|Nicaragua, MIANI098-21|Notonectidae|Nicaragua,MIANI101-21|Notonectidae|Nicaragua, MIANI102-21|Notonectidae|Nicaragua,MIANI097-21|Notonectidae|Nicaragua, MIANI100-21|Notonectidae|Nicaragua,MIANI099-21|Notonectidae|Nicaragua, MIANI094-21|Notonectidae|Nicaragua,MIANI092-21|Notonectidae|Nicaragua, MIANI091-21|Notonectidae|Nicaragua,MIANI089-21|Notonectidae|Nicaragua, MIANI096-21|Notonectidae|Nicaragua,MIANI093-21|Notonectidae|Nicaragua

**Species 20 (support = 1.000)**

MIANI002-21|Farrodes|Nicaragua

**Species 21 (support = 0.915)**

MIANI013-21|Ulmeritoides|Nicaragua,MIANI014-21|Ulmeritoides|Nicaragua, MIANI012-21|Ulmeritoides|Nicaragua,MIANI010-21|Ulmeritoides|Nicaragua, MIANI011-21|Ulmeritoides|Nicaragua

**Species 22 (support = 1.000)**

MIANI121-21|Enallagma|Nicaragua

**Species 23 (support = 1.000)**

MIANI082-21|Gerridae|Nicaragua

**Species 24 (support = 1.000)**

MIANI025-21|Cabecar serratus|Nicaragua,MIANI022-21|Cabecar serratus|Nicaragua,

MIANI024-21|Cabecar serratus|Nicaragua

**Species 25 (support = 1.000)**

MIANI116-21|Neoerythromma cultellatum|Nicaragua

**Species 26 (support = 0.893)**

MIANI041-21|Chironomidae|Nicaragua,MIANI043-21|Chironomidae|Nicaragua

**Species 27 (support = 0.959)**

MIANI049-21|Chironomidae|Nicaragua,MIANI039-21|Chironomidae|Nicaragua

**Species 28 (support = 0.916)**

MIANI066-21|Tabanus|Nicaragua,MIANI074-21|Tabanus|Nicaragua, MIANI069-21|Tabanus|Nicaragua,MIANI071-21|Tabanus|Nicaragua,MIANI075-21|Tabanus|Nicaragua, MIANI068-21|Tabanus|Nicaragua,MIANI065-21|Tabanus|Nicaragua,MIANI067-21|Tabanus|Nicaragua, MIANI077-21|Tabanus|Nicaragua,MIANI070-21|Tabanus|Nicaragua,MIANI076-21|Tabanus|Nicaragua, MIANI072-21|Tabanus|Nicaragua

**Species 29 (support = 1.000)**

MIANI073-21|Chrysops mexicanus|Nicaragua

**Species 30 (support = 0.988)**

MIANI107-21|Veliidae|Nicaragua,MIANI108-21|Veliidae|Nicaragua,

MIANI083-21|Rheumatobates|Nicaragua

**Species 31 (support = 0.491)**

MIANI034-21|Baetidae|Nicaragua,MIANI035-21|Baetidae|Nicaragua

**Species 32 (support = 0.956)**

MIANI027-21|Baetis|Nicaragua,MIANI028-21|Baetis|Nicaragua,

MIANI031-21|Baetis|Nicaragua

**Species 33 (support = 1.000)**

MIANI026-21|Baetidae|Nicaragua

**Species 34 (support = 0.963)**

MIANI051-21|Simulium pulverulentum|Nicaragua, MIANI056-21|Simulium pulverulentum|Nicaragua,MIANI061-21|Simulium pulverulentum|Nicaragua, MIANI055-21|Simulium pulverulentum|Nicaragua,MIANI059-21|Simulium pulverulentum|Nicaragua, MIANI053-21|Simulium pulverulentum|Nicaragua,MIANI054-21|Simulium pulverulentum|Nicaragua, MIANI060-21|Simulium pulverulentum|Nicaragua,MIANI052-21|Simulium pulverulentum|Nicaragua, MIANI064-21|Simulium pulverulentum|Nicaragua,MIANI057-21|Simulium pulverulentum|Nicaragua, MIANI058-21|Simulium pulverulentum|Nicaragua,MIANI062-21|Simulium pulverulentum|Nicaragua

**Species 35 (support = 1.000)**

MIANI063-21|Simulium metallicum|Nicaragua

**Species 36 (support = 0.372)**

MIANI128-21|Dythemis|Nicaragua,MIANI127-21|Dythemis|Nicaragua, MIANI129-21|Dythemis|Nicaragua

**Species 37 (support = 1.000)**

MIANI123-21|Orthemis|Nicaragua

**Species 38 (support = 1.000)**

MIANI030-21|Camelobaetidius|Nicaragua

**Species 39 (support = 1.000)**

MIANI029-21|Camelobaetidius|Nicaragua

**Species 40 (support = 1.000)**

MIANI047-21|Chironomidae|Nicaragua

**Species 41 (support = 1.000)**

MIANI042-21|Chironomidae|Nicaragua

**Species 42 (support = 1.000)**

MIANI038-21|Chironomidae|Nicaragua

**Species 43 (support = 1.000)**

MIANI187-21|Scirtidae|Nicaragua

**Species 44 (support = 0.628)**

MIANI156-21|Chimarra|Nicaragua,MIANI155-21|Chimarra|Nicaragua, MIANI164-21|Chimarra|Nicaragua

**Species 45 (support = 0.957)**

MIANI158-21|Chimarra acuta|Nicaragua,MIANI152-21|Chimarra acuta|Nicaragua, MIANI159-21|Chimarra acuta|Nicaragua,MIANI157-21|Chimarra acuta|Nicaragua

**Species 46 (support = 0.478)**

MIANI006-21|Thraulodes|Nicaragua,MIANI005-21|Thraulodes|Nicaragua

**Species 47 (support = 1.000)**

MIANI007-21|Thraulodes pacaya|Nicaragua,MIANI001-21|Thaulodes pacaya|Nicaragua, MIANI009-21|Thraulodes pacaya|Nicaragua,MIANI004-21|Thraulodes pacaya|Nicaragua, MIANI008-21|Thraulodes pacaya|Nicaragua,MIANI003-21|Thraulodes pacaya|Nicaragua

**Species 48 (support = 1.000)**

MIANI086-21|Trepobates|Nicaragua

**Species 49 (support = 0.172)**

MIANI085-21|Trepobates trepidus|Nicaragua,MIANI087-21|Trepobates trepidus|Nicaragua, MIANI088-21|Trepobates trepidus|Nicaragua,MIANI080-21|Trepobates trepidus|Nicaragua, MIANI084-21|Trepobates trepidus|Nicaragua,MIANI081-21|Trepobates trepidus|Nicaragua, MIANI079-21|Trepobates trepidus|Nicaragua

**Species 50 (support = 1.000)**

MIANI048-21|Rheotanytarsus|Nicaragua

**Species 51 (support = 1.000)**

MIANI037-21|Tanytarsus pandus|Nicaragua

**Species 52 (support = 1.000)**

MIANI183-21|Scirtidae|Nicaragua

**Species 53 (support = 0.917)**

MIANI186-21|Scirtidae|Nicaragua,MIANI191-21|Scirtidae|Nicaragua, MIANI190-21|Scirtidae|Nicaragua,MIANI184-21|Scirtidae|Nicaragua, MIANI180-21|Scirtidae|Nicaragua,MIANI189-21|Scirtidae|Nicaragua, MIANI181-21|Scirtidae|Nicaragua,MIANI188-21|Scirtidae|Nicaragua, MIANI182-21|Scirtidae|Nicaragua,MIANI185-21|Scirtidae|Nicaragua, MIANI192-21|Scirtidae|Nicaragua

**Species 54 (support = 0.971)**

MIANI117-21|Argia tezpi|Nicaragua,MIANI118-21|Argia tezpi|Nicaragua

**Species 55 (support = 0.372)**

MIANI120-21|Argia oenea|Nicaragua,MIANI119-21|Argia oenea|Nicaragua, MIANI115-21|Argia oenea|Nicaragua

**Species 56 (support = 0.980)**

MIANI132-21|Gomphidae|Nicaragua,MIANI133-21|Gomphidae|Nicaragua, MIANI135-21|Gomphidae|Nicaragua,MIANI134-21|Gomphidae|Nicaragua,MIANI131-21|Gomphidae|Nicaragua,MIANI137-21|Gomphidae|Nicaragua

**Species 57 (support = 0.895)**

MIANI138-21|Erpetogomphus|Nicaragua,MIANI130-21|Erpetogomphus|Nicaragua, MIANI136-21|Erpetogomphus|Nicaragua

**Species 58 (support = 1.000)**

MIANI109-21|Rhagovelia|Nicaragua

**Species 59 (support = 1.000)**

MIANI110-21|Rhagovelia|Nicaragua

**Species 60 (support = 1.000)**

MIANI106-21|Rhagovelia|Nicaragua

**Species 61 (support = 0.470)**

MIANI104-21|Rhagovelia|Nicaragua,MIANI105-21|Rhagovelia|Nicaragua

**Species 62 (support = 1.000)**

MIANI032-21|Americabaetis|Nicaragua

**Species 63 (support = 1.000)**

MIANI033-21|Americabaetis|Nicaragua

**Species 64 (support = 1.000)**

MIANI126-21|Brechmorhoga|Nicaragua

**Species 65 (support = 0.952)**

MIANI122-21|Perithemis|Nicaragua,MIANI124-21|Perithemis|Nicaragua

**Species 66 (support = 1.000)**

MIANI125-21|Perithemis|Nicaragua

**Species 67 (support = 1.000)**

MIANI021-21|Tricorythodes|Nicaragua

**Species 68 (support = 0.158)**

MIANI020-21|Tricorythodes|Nicaragua,MIANI017-21|Tricorythodes|Nicaragua, MIANI016-21|Tricorythodes|Nicaragua,MIANI018-21|Tricorythodes|Nicaragua, MIANI019-21|Tricorythodes|Nicaragua

**Species 69 (support = 0.493)**

MIANI113-21|Veliidae|Nicaragua,MIANI111-21|Veliidae|Nicaragua

**Species 70 (support = 0.951)**

MIANI112-21|Veliidae|Nicaragua


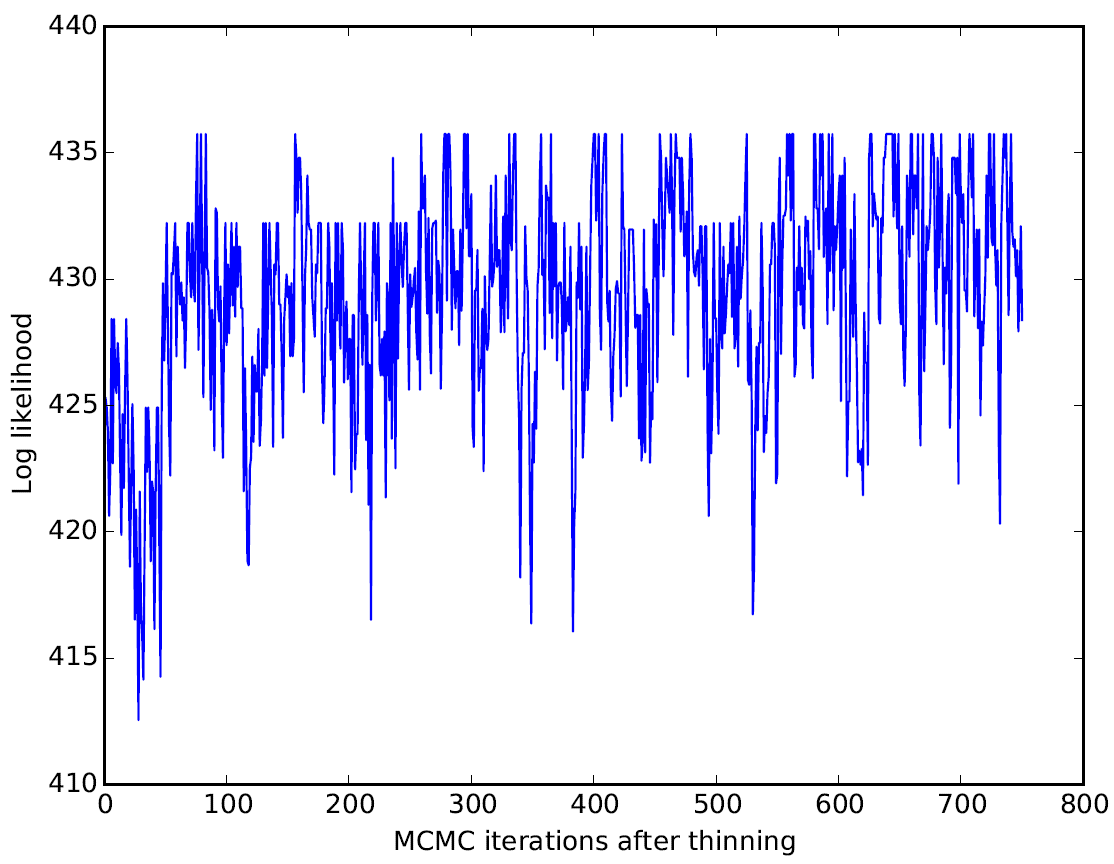

Supplement: Supplementary file 2 — Appendix S2. [file ECE3-12-e9487-s002.docx]
